# Computational investigation of epithelial cell dynamic phenotype in vitro

**DOI:** 10.1186/1742-4682-6-8

**Published:** 2009-05-28

**Authors:** Sean HJ Kim, Sunwoo Park, Keith Mostov, Jayanta Debnath, C Anthony Hunt

**Affiliations:** 1 UCSF/UC Berkeley Joint Graduate Group in Bioengineering, University of California, Berkeley, California 94720, USA; 2 Department of Bioengineering and Therapeutic Sciences, University of California, San Francisco, California 94143, USA; 3 Department of Anatomy, University of California, San Francisco, California 94143, USA; 4 Department of Pathology, University of California, San Francisco, California 94143, USA

## Abstract

**Background:**

When grown in three-dimensional (3D) cultures, epithelial cells typically form cystic organoids that recapitulate cardinal features of in vivo epithelial structures. Characterizing essential cell actions and their roles, which constitute the system's dynamic phenotype, is critical to gaining deeper insight into the cystogenesis phenomena.

**Methods:**

Starting with an earlier in silico epithelial analogue (ISEA1) that validated for several Madin-Darby canine kidney (MDCK) epithelial cell culture attributes, we built a revised analogue (ISEA2) to increase overlap between analogue and cell culture traits. Both analogues used agent-based, discrete event methods. A set of axioms determined ISEA behaviors; together, they specified the analogue's operating principles. A new experimentation framework enabled tracking relative axiom use and roles during simulated cystogenesis along with establishment of the consequences of their disruption.

**Results:**

ISEA2 consistently produced convex cystic structures in a simulated embedded culture. Axiom use measures provided detailed descriptions of the analogue's dynamic phenotype. Dysregulating key cell death and division axioms led to disorganized structures. Adhering to either axiom less than 80% of the time caused ISEA1 to form easily identified morphological changes. ISEA2 was more robust to identical dysregulation. Both dysregulated analogues exhibited characteristics that resembled those associated with an in vitro model of early glandular epithelial cancer.

**Conclusion:**

We documented the causal chains of events, and their relative roles, responsible for simulated cystogenesis. The results stand as an early hypothesis–a theory–of how individual MDCK cell actions give rise to consistently roundish, cystic organoids.

## Background

How single cells proliferate and organize into liquid filled cysts, or acini, is a central question in epithelial morphogenesis and cancer research. Epithelial cells in tissues engage an array of activities to attain acinar structures [1]. The same is true in cultures. When grown embedded in 3D culture, epithelial cells such as Madin-Darby canine kidney (MDCK) cells develop stereotypical cystic organoids by mechanisms that can differ depending on culture conditions [2]. When manipulated or exposed to certain factors, these organoids and composing cells can exhibit phenotypic attributes that are reminiscent of pre-cancerous or cancerous tissues [3]. While MDCK culture models are orders of magnitude simpler than epithelial cells in tissues, they provide an appropriate physiological environment to study epithelial cyst development, function, and pathology. However, they too are complex dynamic systems that have proven challenging to understand.

The emergence of stable organoid structures is the cumulative consequence of individual cell actions: the system's dynamic phenotype. Disruption of one or more of these actions can cause potentially pathologic changes. Little is known about the varying cell mechanisms and activities that engage in different stages of cystogenesis and how they contribute to the process. A strategy to understanding the phenomena must include classifying those essential cell actions and tracing their relative use and roles as the process unfolds. With time-lapse, microscopy images alone, it can be difficult to ascertain what cell actions are responsible for the observed structure transformations.

**Figure 1:**
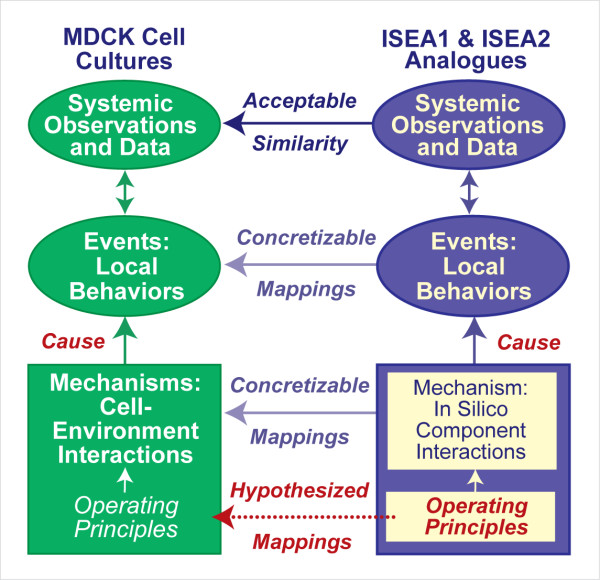
**Relationships between analogues and MDCK cultures**. To distinguish simulation components and characteristics from in vitro counterparts, we use small caps when referring to the former. An in silico epithelial analogue (ISEA) is comprised of autonomous CELL components interacting with adjacent CELLS and environment components. Interactions are governed by a set of axiomatic operating principles (rules). For each environment circumstance a CELL can encounter, there is a corresponding axiom. A clear mapping exists between ISEA components (CELL states and environment components) and in vitro counterparts. Following execution, interacting components cause local and systemic behaviors. Measures of CELL and system behaviors (growth rates, structure type, etc.) are the in silico attributes. Validation occurs when a set of ISEA attributes is measurably similar to a corresponding, prespecified set of in vitro attributes. Upon validation, we can hypothesize that a semiquantitative mapping exists between ISEA events and in vitro events, and that the set of in silico operating principles has a biological counterpart.

Computational methods detailed herein represent an additional, synergistic approach to gain the much-needed insight. The approach used [4-6] is an example of executable biology [7,8]. We used in silico epithelial analogues (ISEAs) that have undergone validation against a targeted set of MDCK epithelial cell attributes. As discussed in [9], the attributes targeted by the earlier analogue (ISEA1) were selected to reflect essential MDCK cell behaviors in cultures but for simplicity, the list excluded other MDCK attributes. Our goal was to improve ISEA1 in stages to achieve increased phenotype overlap between the revised analogue (ISEA2) and MDCK cell cultures. To keep improvement parsimonious, we expanded the original list by one additional attribute: all stable cyst structures must have a convex contour without irregular margins or dimples. Unlike its referent, ISEA1 frequently produced cyst structures having irregular shapes. Through exploratory simulations discussed below, we discovered and added one new cell action to achieve the additional attribute. The mappings from in silico components, their spatial arrangement, their mechanisms of interactions, and system-level attributes to their in vitro counterparts (Figure 1) improved following that refinement.

Cell biologists compare and contrast the growth characteristics of different, related epithelial cell lines in part to better understand how and where their behaviors differ or are similar. That knowledge can be used to make better inferences about referent cell behaviors in vivo. A proven wet-lab approach is to design and conduct experiments to test hypotheses about cell line responses to interventions, such as blocking a signaling pathway or a cell surface receptor. Analogous methods must be used to study and compare phenotypic attributes of in silico analogues, such as ISEA1 and ISEA2. In addition, study of analogue responses to interventions improves insight into MDCK morphogenesis. Differences in morphological and dynamic phenotype, or lack thereof, between two analogues could shed additional insight on those of the referent [10]. With that in mind, we compared ISEA1 and ISEA2 behaviors to understand how specific mechanistic changes alter their morphogenetic attributes.

**Figure 2:**
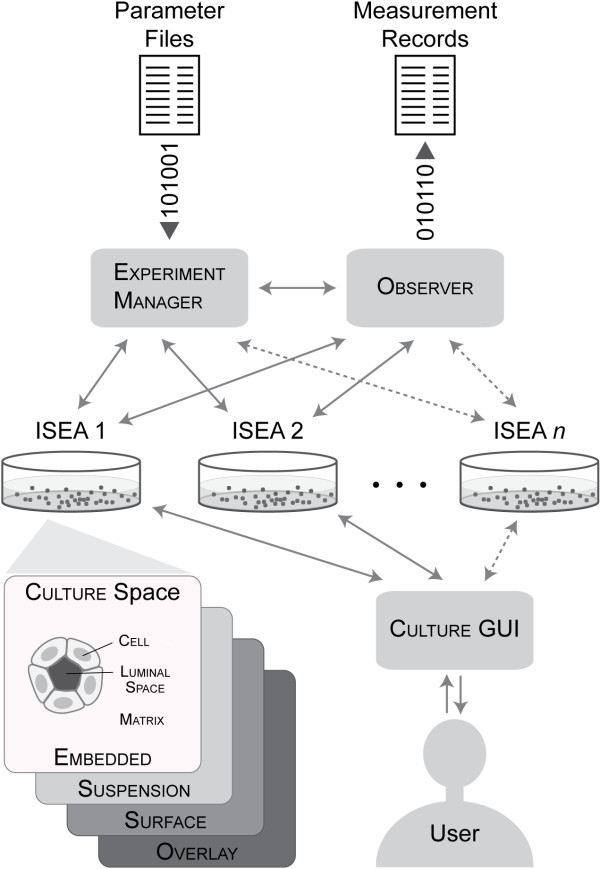
**ISEA components and system architecture**. The in silico system consists of a CULTURE and framework components. MDCK cell cultures and ISEAs are both composite systems. A CULTURE represents one in vitro cell culture. It is a composite of three object types: CELLS, MATRIX, and free space. A hexagonal grid provides the space (CULTURE space) within which components interact. CELLS are quasi-autonomous agents whose actions are driven by their internal logic and a set of axiomatic operating principles. MATRIX maps to extracellular matrix, and FREE SPACE maps to aqueous material (e.g., cyst lumen) devoid of cells and matrix. Both are passive objects. ISEA1 [9] validated for basic, target attributes of four different cell culture types: embedded, suspension, surface, and overlay. ISEA1 was revised to ISEA2, which validated for an expanded set of target attributes. The framework provides components and methods to enable semi-automatic experimentation and analysis. EXPERIMENT MANAGER is the experiment control agent. It prepares parameter files, manages experiments, and processes data. OBSERVER is a module that automatically conducts and records measurements on CULTURE. CULTURE GUI provides a graphical interface to visualize and interactively probe CULTURE during execution.

ISEA1 and ISEA2 used sets of rules in the form of axioms for determining CELL action based on CELL neighbor type and configuration. Each simulation cycle, each CELL assessed the current arrangement of neighbors, selected the corresponding axiom, and then executed that axiom's action. By adhering strictly to their axioms, both analogues achieved their respective set of targeted attributes. Are actions of MDCK cells in cultures (and epithelial cells in general) so rigidly choreographed? How tightly must ISEA adhere to its operating principles before aspects of phenotype become measurably abnormal? We gained insight into plausible answers by systematically relaxing two, key ISEA actions and exploring in detail the phenotypic consequences. One action mapped to anoikis, a specific category of cell death. The other involved directed placement of an ISEA daughter cell, a form of oriented cell division. The ISEA1 phenotype was quite sensitive to dysregulating the two actions: engaging in either action less than 80% of the time caused easily detected phenotypic changes. Interestingly, ISEA2 was more robust to identical disruptions. Both ISEA1 and ISEA2 exhibited phenotypes that resembled those associated with an in vitro model of early glandular epithelial cancer. To the extent that the in silico-to-in vitro mappings in Figure 2 are valid, ISEA2's operating principles and dynamic phenotype stand as hypotheses of their MDCK counterparts in cell culture.

## Methods

### In vitro cell culture experiments

Full details of the original MDCK cell culture experiments are provided in [11]. Briefly, MDCK cells were triturated into single-cell suspensions in type I collagen gel. Cells were grown for 7–10 d until cysts with lumina formed. For immunofluorescence staining of cysts, samples were incubated with primary antibodies overnight, followed by an overnight incubation with fluorescent dye-labeled secondary antibodies. To quantitate cyst polarity, cysts were stained for gp135 (apical surface), β-catenin (basolateral surface) and nuclei, and then visualized using a confocal microscope.

### In silico experimentation framework

ISEA1 and ISEA2 are discrete event [12], agent-based [13] systems that comprise the core analogue and system-level components for experimentation and analysis (Figure 2). Because ISEA2 is based on ISEA1, both share a common design, and their experiment features overlap significantly (discussed below). Before moving forward with model refinement and experimentation, implementation redundancies of ISEA1 and ISEA2 were removed. We revised the existing framework to enable simulation of multiple, somewhat different CELL analogue types. ISEA1 was ported and revalidated within the new framework prior to ISEA2 development. To clearly distinguish ISEA components and processes from their in vitro counterparts, hereafter we use small caps when referring the former.

We created system-level components including EXPERIMENT MANAGER, OBSERVER, and CULTURE graphical user interface (GUI) to enable semi-automated experimentation and analysis. EXPERIMENT MANAGER, the top-level system component, is an agent that provides experiment protocol functions and specifications. The specifications define the mode of experimentation and the system's parameter vector. Experiments can be conducted in default, visual, or batch modes. Batch mode enables automatic construction and execution of multiple experiments, as well as processing and analysis of recorded measurements. Based on user-defined specifications, EXPERIMENT MANAGER automatically generates a set of parameter files and executes a batch of experiments, each corresponding to a different parameter file. After completion of all experiments, basic analytic operations collect and summarize data. OBSERVER is responsible primarily for recording measurements. At the end of every simulation cycle, OBSERVER scans the CULTURE internals and performs measurements. The measurements are recorded as time series vectors. At simulation's end, data are written to a set of files for analytic processing by EXPERIMENT MANAGER. CULTURE GUI provides a visualization console, which can be used interactively to start or pause a simulation and to access live states of CULTURE grid content. Using CULTURE GUI functionalities, OBSERVER can capture time-lapse CULTURE images and store them in multiple formats for post-processing.

### ISEA1 and ISEA2 designs are agent-based and object-oriented

Detailed descriptions of ISEA1 design features, and development methods, are available in [9]. ISEA2 design uses similar features, which have been refined to meet study requirements. An abridged description follows. The referent in vitro cell culture was conceptually abstracted into four components: cells, media containing matrix (matrix hereafter), matrix-free media (free space hereafter), and a space to contain them. Discrete software objects with eponymous names represent those four essential cell culture components: CELL, MATRIX, FREE SPACE, and CULTURE. MATRIX and FREE SPACE are passive objects. A MATRIX object maps to a cell-sized volume of extracellular matrix (ECM). A FREE SPACE object maps to a similarly sized volume of material that is essentially free of cells and matrix elements. FREE SPACE also represents luminal space and non-matrix material in pockets enclosed by cells. The latter are called LUMINAL SPACE when distinction from FREE SPACE is useful. CELLS are quasi-autonomous agents (as agents, they can schedule their own events; they follow their own agenda). They use a set of rules or decision logic to interact with their local environment. A CULTURE is an agent that maps abstractly to a cell culture within one well of a multi-well culture plate. The CULTURE uses a standard two-dimensional (2D) hexagonal grid to provide the space in which its objects reside. The grid has toroidal topologies. For simplicity, each grid position is occupied by one object. That condition can be changed when the need arises.

There is a direct link between the choice of level of detail—granularity—and the list of targeted attributes. Granularity is the extent to which a larger entity is subdivided. There is also a direct link between required mechanistic detail and granularity. We can discover that a cell always (or almost always) executes a particular move when confronted with a specific situation without knowing (or needing to represent) details of how the move was accomplished. Our goal has been to first discover plausible cell-level mechanistic details that account for a variety of targeted attributes; cell size is thus a logical granularity level. We can then explore more detailed (fine-grained) explanations for how a particular mechanistic detail was enabled, because a coarse-grained component can be replaced by a finer-grained component when that is needed. A more coarse-grained mechanism that can account for targeted attributes is preferred over a more detailed mechanism because the coarse-grained mechanism is simpler. The parsimony guideline is to prefer the simpler explanation of the facts (the targeted attributes).

### ISEA execution protocol

A CULTURE has base methods that are called automatically at a simulation's start and end. The start function initializes the grid and CULTURE components, CELLS, MATRIX, and FREE SPACE. Simulation starts upon completion of that process. As execution advances, the event schedule is stepped for a number of simulation cycles or until a stop signal is produced. At simulation's end, the CULTURE finish function closes open files and clears the system.

**Figure 3:**
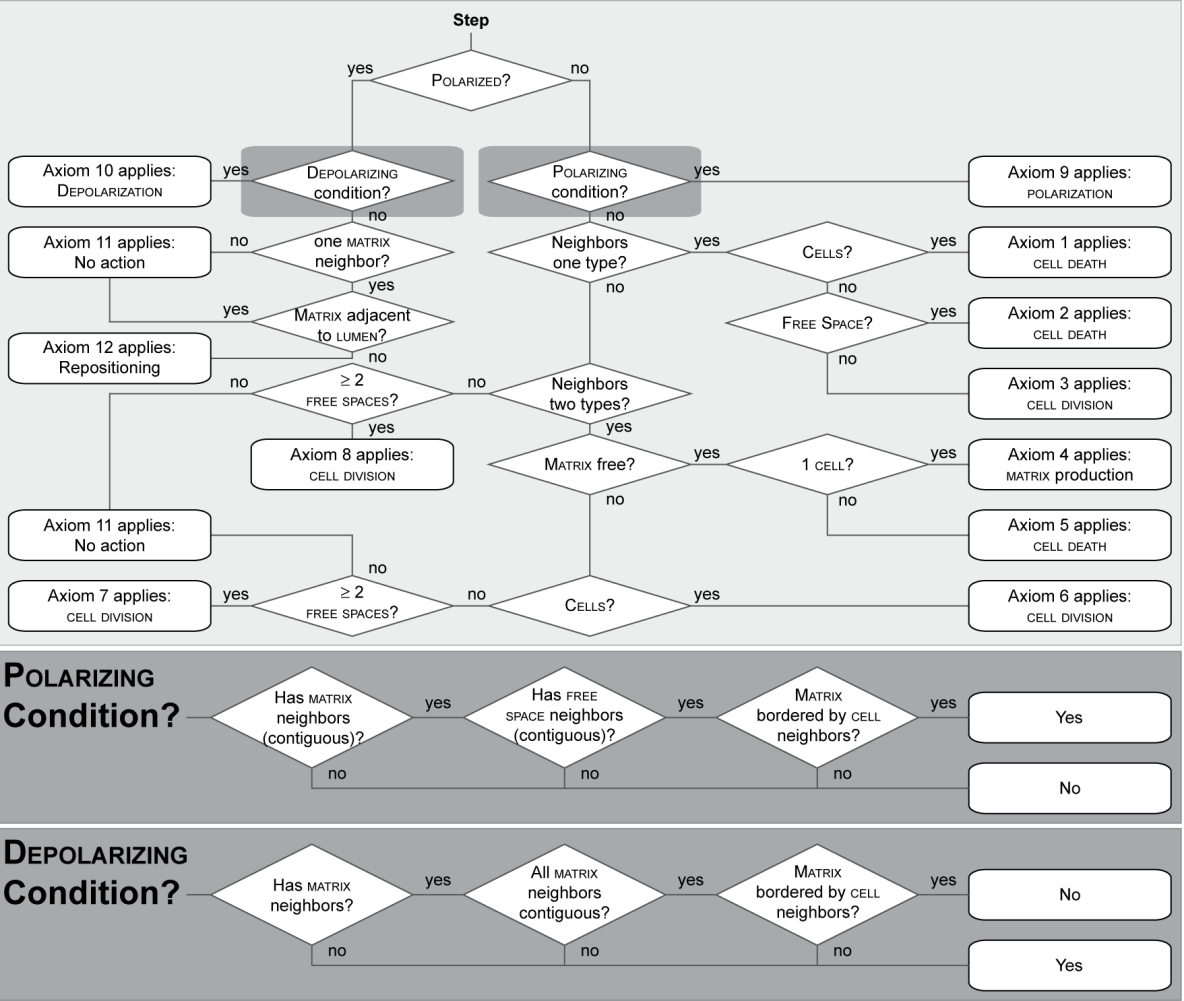
**ISEA2 CELL decision logic and axiomatic operating principles**. Simulation time advances by simulation cycles. During a simulation cycle, every CULTURE component is given an opportunity to update. Every CELL in a pseudo-random order decides what action to take based on its internal state (POLARIZED or UNPOLARIZED) and the composition of its adjacent neighborhood. Actions available to UNPOLARIZED CELLS are: DIE, create a new CELL, produce MATRIX, POLARIZE, and do nothing. POLARIZED CELLS have three options: DEPOLARIZE, reposition, or do nothing. At every decision point, the CELL uses the diagrammed logic to select and execute just one action. We iteratively refined ISEA1 to ISEA2. It consistently produced convex, cystic structures in addition to achieving the original set of targeted attributes.

Simulation time advances discretely, and is maintained by a master event schedule. Event ordering within a simulation cycle is pseudo-random. Having objects update pseudo-randomly simulates the parallel operation of cells in culture and the nondeterminism fundamental to living systems, while building in a controllable degree of uncertainty. Within a simulation cycle, each CELL in pseudo-random order is given an opportunity to interact with adjacent objects in its environment and, if required, undertake an action. Every CELL uses the same step function. A set of CELL axioms (Figure 3) determines all CELL actions. A CELL selects just one axiom and corresponding action during each simulation cycle.

### Axiomatic operating principles

An agent has rules and protocols for interacting with external components. Rules can take any form. We elected to have all rules take the form of axioms. We use the term "axiom" to reinforce an idea that our computational model is a mathematical, formal system and that analogue execution is a form of deduction from the original axioms or assumptions explicitly programmed into the model. An axiom specifies a precondition and corresponding action. We specified what we judged to be a minimal set of action options: replace an adjacent non-CELL object with a CELL copy, DIE (vanish) and leave behind a LUMINAL SPACE, create MATRIX, destroy an adjacent non-CELL object and move to that location leaving behind a LUMINAL SPACE, POLARIZE, DEPOLARIZE, and do nothing. For any precondition, only one action option was executed.

ISEA1 had eleven axiomatic operating principles that enabled the analogue to validate against its initial targeted attributes. For convenience, the final ISEA1 axioms are summarized as follows. The precondition applies to the six objects adjacent to each CELL.

1. All neighbors are CELLS: DIE (delete self) and leave behind a LUMINAL SPACE.

2. All neighbors are LUMINAL SPACE: DIE and leave behind a LUMINAL SPACE.

3. All neighbors are MATRIX: replace a randomly selected MATRIX with a CELL copy.

4. Neighbors comprise one CELL and LUMINAL SPACES: add MATRIX between self and the adjoining CELL.

5. Neighbors comprise at least two CELLS and LUMINAL SPACES, but no MATRIX: DIE (undergo ANOIKIS) and leave behind a LUMINAL SPACE.

6. Neighbors comprise at least one CELL and MATRIX: create a CELL copy; the copy replaces any MATRIX that maximizes its number of CELL neighbors.

7. Neighbors comprise at least two LUMINAL SPACES and MATRIX: create a CELL copy; the copy replaces any LUMINAL SPACE that adjoins MATRIX.

8. Neighbors comprise CELLS, MATRIX, and at least two adjacent LUMINAL SPACES: create a CELL copy; the copy replaces any LUMINAL SPACE neighbor that adjoins MATRIX and LUMINAL SPACE.

9. Two CELL neighbors are separated on one side by MATRIX and on the other side by LUMINAL SPACE: POLARIZE.

10. A POLARIZED CELL has noncontiguous MATRIX neighbors: revert to NONPOLARIZED CELL state.

11. None of the preceding preconditions has been met: do nothing; CELL mandates achieved.

Detailed descriptions of supporting biological evidence and assumptions made for ISEA1 CELL axioms are provided in [9]. Briefly, CELL DEATH axioms (Axioms 1, 2, and 5) were based on a general biological principle that cells, such as epithelial cells, undergo a process of cell death within some interval after detaching from ECM [14,15]. That behavior is observed in MDCK cell cultures [2,16]. Axiom 4, which dictates MATRIX deposition between two adjacent CELLS, was specified based on observations that some matrix is produced de novo between two adhering MDCK cells in suspension culture [17]. A CELL DIVISION axiom, Axiom 3, follows from experimental observations that, when embedded in matrix, single MDCK cells proliferate [11,16]. Other CELL DIVISION axioms, Axioms 6, 7, and 8, follow from a similar, general principle that epithelial cells proliferate when they adhere to ECM and tend do so in arrangements that maximize intercellular contact [18,19]. CELL POLARIZATION axioms, Axioms 9 and 10, reflect in vitro observations on MDCK cell polarity [2,18]. Axiom 11 applied when the CELL achieved mandates that map to the three-surfaces principle articulated in [1,18].

Starting with the ISEA1 axioms, we devised, tested, and iteratively refined candidate axioms to enable the CELLS to consistently develop CYSTS with smooth margins and a convex shape (in the hexagonal grid representation), while validating for the targeted attributes described in [9]. At each step, variations of an axiom were tested, and those that moved the analogue closer to validation were selected for further refinement. In its validated form, ISEA2 used Axioms 1–10 from ISEA1 without change. However, ISEA1's Axiom 11 was replaced by the following two axioms.

11. Neither the preceding nor the following preconditions have been met: do nothing; CELL mandates achieved.

12. A POLARIZED CELL confirms that Axiom 9 precondition is met and has only one MATRIX neighbor: the POLARIZED CELL deletes the adjacent MATRIX, moves to its location, and leaves behind a LUMINAL SPACE.

The revised axioms diagrammed in Figure 3 and summarized in Table 1 represent what we determined as a minimal change that was required for final validation. Revisions that were more elaborate also enabled those ISEAs to achieve the target attributes. However, they were rejected because they were not parsimonious. The final validation required that > 98% of the CYSTS formed during 50 simulation cycles in 100 Monte Carlo simulations must have a roundish, convex shape (visually inspected). We determined by visual inspection that convex CYSTS had no dimples or irregular margins. Manual inspection of the ISEA CYSTS sufficed for this study's purposes. However, we will need algorithmic metrics to expedite and automate analyzing and quantifying CYST convexity.

**Table 1:** ISEA CELL axioms and consequences of dysregulated CELL actions.

Axiom	Precondition	Action	Dysregulated action	Observed morphological changes
1	CELLS only	DIE	Do nothing	None (*p *> 0); unchecked growth (*p *= 0)

2	LUMINAL SPACE only	DIE	Do nothing	None (*p *≥ 0)

3	MATRIX only	DIVIDE non-directionally	Do nothing	None (*p *≥ 0)

4	1 CELL and LUMINAL SPACES; no MATRIX	Produce and deposit MATRIX	Do nothing	None (*p *≥ 0)

5	≥ 2 CELLS and LUMINAL SPACE; no MATRIX	DIE	Do nothing	Increased CELL population; nested CELL CLUSTERS in CYST LUMEN (*p *< 1)

6	≥ 1 CELL and MATRIX; no LUMINAL SPACE	DIVIDE directionally	DIVIDE in a random direction	Increased CELL population; nested CELL CLUSTERS in CYST LUMEN (*p *< 1)

7	MATRIX and ≥ 2 LUMINAL SPACES; no CELLS	DIVIDE directionally	DIVIDE in a random direction	None (*p *≥ 0)

8	CELLS, MATRIX, and ≥ 2 adjacent LUMINAL SPACES	DIVIDE directionally	N/A	N/A

9	2 CELLS, MATRIX, and LUMINAL SPACE; POLARIZING condition*	POLARIZE	N/A	N/A

10	DEPOLARIZING condition^†^	DEPOLARIZE	N/A	N/A

11	All other configurations	Do nothing	N/A	N/A

12	POLARIZING condition; 1 MATRIX	Move and replace the neighboring MATRIX	Do nothing	Frequently irregular, nonconvex CYST shape (*p *< 1)

Each axiom's precondition takes into consideration the six objects adjacent to the decision-making CELL. In dysregulated state, the normal CELL action applies with probability p; the dysregulated CELL action applies with probability 1 - p. Exploration of Axioms 8–11 was beyond the scope of this study. Axiom 12 is used only by ISEA2.

* Two CELL neighbors separate MATRIX on one side from LUMINAL SPACE on the other side. ^†^A POLARIZED CELL has noncontiguous MATRIX neighbors.

### Operational disruption of ISEA CELL axioms

We implemented a method to disrupt selectively the operation of individual CELL axioms. We added a parameter, *p*, for each axiom. It controlled the probability of the decision-making CELL electing to follow the axiom when its precondition applied. Parameter values ranged from 0 to 1 inclusively. A parameter value = 1 corresponded to 100% adherence. Setting it to zero completely blocked the prescribed action and, as specified, dictated an alternate action. An additional control was added to allow the CELL to draw a pseudo-random number (PRN) from the standard uniform distribution at each decision point. The axiom's prescribed action was followed only when the PRN was ≤ the probability threshold set by its parameter.

We considered, and used when applicable, alternative actions that map to plausible in vitro cell actions occurring in a dysregulated state (Table 1). Axioms 1, 2, and 5 governed CELL DEATH; a reasonable alternative was to remain ALIVE (i.e., do nothing). Axiom 3 dictated non-directional CELL DIVISION; its alternate action was to do nothing (i.e., prevent REPLICATION). We also assigned the alternate action of 'do nothing' to Axiom 4 (MATRIX production). Several dysregulated action options were available for Axiom 6 (directed CELL DIVISION). One was to do nothing, effectively suppressing CELL DIVISION. Another was DISORIENTED CELL DIVISION, positing the CELL copy in a random direction without regard for the number of CELL neighbors. We elected to use the latter, for which adequate, supportive biological information is available [20-23]. Axiom 7, which dictated CELL DIVISION, had available the same alternative action options. Axiom 8 (CELL DIVISION or POLARIZATION) had a precondition comprising all three component types (CELL, MATRIX, and LUMINAL SPACE), which presented many plausible action options. One option was preventing CELL DIVISION; another was to allow the CELL to DIVIDE non-directionally as described above. Another option was to initiate POLARIZATION. The remaining axioms, Axioms 9–12, posed a similar problem of having many plausible action options. Because no wet-lab experimental insight was available to narrow the options, we elected to defer investigation of those axioms until more information becomes available.

### Simulation experiment design

The following describes design and execution of ISEA1 and ISEA2 simulation experiments. First, the top-level system component, EXPERIMENT MANAGER, was initialized. Next, EXPERIMENT MANAGER created a new CULTURE and filled its grid with MATRIX. The grid width and height were set to 100. CULTURE initialized a PRN generator with a seed set to the system's clock. A new seed was used to initialize the CULTURE'S PRN generator at the start of each simulation. Pseudo-random seeds were generated from the CULTURE'S PRN generator to initialize those used by CELLS. Following CULTURE grid setup, one CELL was placed at the center of the CULTURE grid, replacing an existing MATRIX object. The simulation started when the initialization of the CULTURE contents was completed. Each simulation experiment comprised 100 Monte Carlo (MC) runs. Each MC run was executed for 50 simulation cycles. At simulation's end, the recorded measurements were written to files and the CULTURE was destroyed. A new CULTURE was created for each repetition.

### Implementation tools

The model framework was implemented using MASON, a multi-agent, discrete event simulation library, coded in Java [24]. Batch simulation experiments were performed on a small-scale Beowulf cluster system. For model development, testing, and analysis, we used personal computers. Computer codes and project files are available at .

## Results

**Figure 4:**
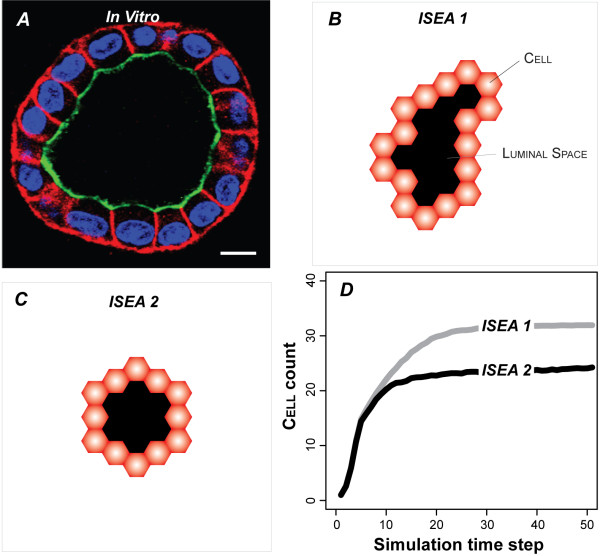
**Cyst growth in simulated and in vitro MDCK cell culture**. (A) MDCK cells grown in 3D matrix form lumen-enclosing cystic organoids surrounded by a layer of polarized cells. Cells composing the cysts maintain three surface types: apical (green), basal and lateral (red). Note the roundish contour typical of MDCK cysts. For growth and staining details, see [11]. Bar: 10 μm. (B) ISEA1 CELLS in EMBEDDED condition produced stable, cystic structures enclosing LUMINAL SPACE; all CELLS were POLARIZED (red). Many CYSTS like the one shown, had irregular, non-convex shapes unlike their in vitro counterpart. (C) ISEA2 CELLS under the same condition also developed stable CYSTS; almost all stabilized CYSTS had convex shapes. Note that a hexagonal CYST within the hexagonally discretized space maps to a roundish cross-section through a MDCK cyst in vitro. (D) ISEA2 CELLS formed CYSTS that tended to be smaller than those of ISEA1 (average 27 vs 31 CELLS per CYST). The CELL count represents mean values after 50 simulation cycles of 100 Monte Carlo runs.

To validate against the targeted attributes, a single CELL was placed in CULTURE space, surrounded by MATRIX. As simulation progressed, the CELL underwent repeated rounds of REPLICATION, followed by LUMINAL SPACE formation and CYST maturation. The LUMINAL SPACE grew as CELLS in the inner region DIED (and vanished) or moved outward. Growth characteristics were similar to those observed in MDCK embedded cultures (Figure 4A). CULTURES always formed stable CYSTS bordered by POLARIZED CELLS (Figure 4B, C). Most ISEA1 CYSTS had irregular shapes. ISEA2 consistently produced CYSTS having a roundish, convex shape (Figure 4C). CYSTS in ISEA2 CULTURES stabilized with fewer CELLS (Figure 4D) than did ISEA1.

For dysregulation experiments, we focused on two critical CELL axioms, Axioms 5 and 6. Axioms 2, 3, 4, and 7, were not critical to CYST formation in EMBEDDED CULTURE (they were critical in other CULTURE conditions, such as monolayer), and were infrequently used, so they were excluded from detailed analysis. Although not essential for EMBEDDED CULTURE, Axiom 4 proved to be an important yet rare event axiom, as discussed below. Disrupting Axiom 8 is not straightforward: if the axiom is not applied, some alternative action must follow from its precondition, and there are many plausible options. We elected not to pursue disruption of Axiom 8 until further insight from wet-lab studies becomes available to narrow options. Disrupting Axiom 1 was straightforward, but the results (not shown) offered no significant insight: CLUSTERS either developed normally into CYSTS for *p *> 0 or grew unchecked as a solid mass when *p *= 0. We expected that outcome because Axiom 1 was required for initial LUMINAL SPACE creation but became nonessential thereafter. On the other hand, Axioms 5 and 6 were essential to CYST formation. Anoikis is a form of cell death that epithelial cells undergo when they lose direct matrix contact [14]. Axiom 5 dictates ANOIKIS. It is the most frequently used CELL DEATH axiom in both ISEA1 and ISEA2. Axiom 6 dictates directed CELL creation (the event maps to selective placement of a daughter cell), and accounts for most of the CELL creation events in both analogues. The in vitro counterparts of Axioms 5 and 6 are centrally implicated in epithelial morphogenesis and carcinogenesis, and have been shown to be important in the context of in vitro cell cultures.

### Dysregulation of Axiom 5 (ANOIKIS)

**Figure 5:**
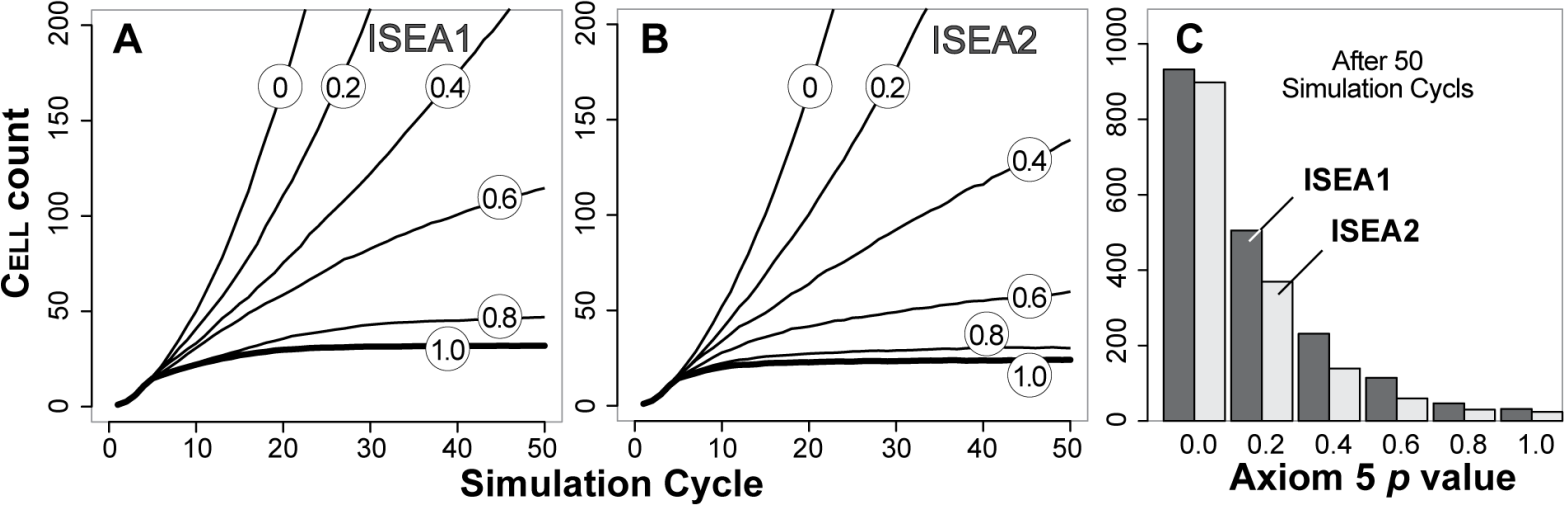
**Dysregulation of Axiom 5 (ANOIKIS) and its effect on ISEA growth and morphology**. Axiom 5 dictates CELL DEATH when the decision-making CELL has in its neighborhood at least two CELLS and LUMINAL SPACE but no MATRIX. CELLS followed Axiom 5 with a parameter-controlled probability, *p*. Otherwise, the Axiom 5 precondition produced no CELL DEATH. Evasion of Axiom 5 changed ISEA1 and ISEA2 growth and structural characteristics in EMBEDDED CULTURES. (A-B) CELL counts at six levels of dysregulation are shown. Values are means of 100 Monte Carlo runs. CELL count increased monotonically with the severity of dysregulation. For ISEA2, the effects were less dramatic for larger *p*. (C) Dysregulation caused a nonlinear increase in both ISEA1 and ISEA2 CELL count measured after 50 simulation cycles.

**Figure 6:**
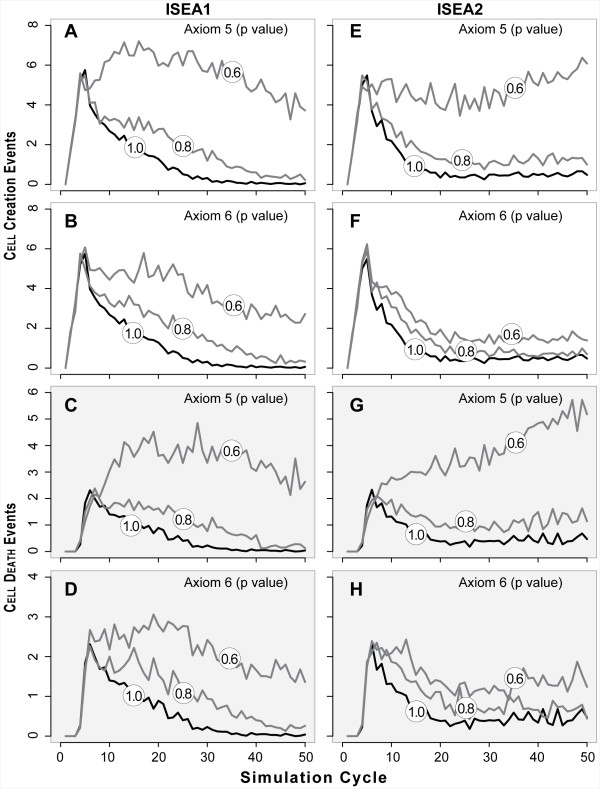
**CELL DEATH and creation events by ISEA1 and ISEA2 with and without dysregulation (two levels) of Axioms 5 and 6**. Values in circles are axiom *p* values.  Left panels (A–D): ISEA1 events. Right panels (E-H): ISEA2 events. Top four panels: CELL creation events. Bottom four panels: CELL DEATH events. Event values are occurrences per simulation averaged over 100 Monte Carlo runs.

In MDCK cultures, apoptosis contributes centrally to lumen formation [2]. Cells in the inner region of the developing structure undergo anoikis. We speculated that if ISEA CELL actions have MDCK counterparts, then the two analogues would exhibit (predict) LUMEN filling when ANOIKIS is compromised. We simulated the condition by disrupting application of Axiom 5. So doing caused aberrant growth (Figure 5) and changed CELL activity patterns (Figure 6). Growth rates increased nonlinearly with increasing dysregulation. ISEA1 was more sensitive to dysregulation at mid-range *p *of 0.4 and 0.6 than was ISEA2. No marked differences were noted at other tested levels. CELL population measurements after 50 simulation cycles reflected changes in growth (Figure 5C). ISEA2 (vs ISEA1) produced structures having fewer CELLS.

**Figure 7:**
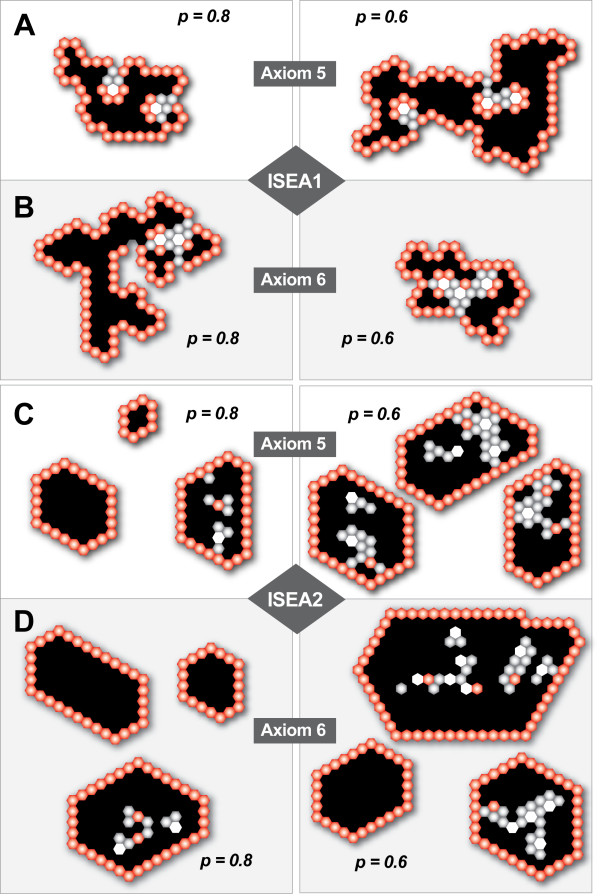
**Typical structures formed by ISEA1 and ISEA2 when Axiom 5 or 6 was dysregulated**. Shown are images of structures formed after 50 simulation cycles for *p *= 0.8 and 0.6. Note that a regular hexagon in 2D hexagonal space maps to a circle in 2D continuous space. Objects: POLARIZED CELL (red), UNPOLARIZED CELL (gray), MATRIX (white), and LUMINAL SPACE (black). (A-B) Shown are examples of structures formed when ISEA1 was dysregulated. (C-D) Shown are examples of structures formed when ISEA2 was dysregulated.

Visual assessment of sample images showed that the CULTURE morphology became irregular with increased dysregulation (Figure 7). Relative to ISEA2, irregularities were more pronounced when ISEA1's Axiom 5 was dysregulated. When CELL creation events outpaced DEATH, small, inverted CYSTS formed and stabilized (through POLARIZATION) within LUMENS. As dysregulation increased, surface irregularities postponed POLARIZATION enabling further CELL creation events and surface expansion. For ISEA2, other factors contributed to LUMEN clearing. The convexity drive (Axiom 12) enabled surface CELLS to POLARIZE sooner. It also retarded inverted CYST formation by CELLS trapped within LUMENS. Trapped CELLS were thus more likely to satisfy the precondition of Axiom 5, even when Axiom 5 was partially dysregulated.

Figure 6 shows how changes in CELL activity patterns accompanied morphology changes for two levels of Axiom 5 dysregulation. ANOIKIS dysregulation changed the occurrence frequencies of axiom preconditions. That change resulted in increased CELL creation events for both ISEA1 and ISEA2. Interestingly, for *p *= 0.8 and 0.6, those changes led to a net increase in CELL DEATH events. For ISEA1, many of the additional CELL creation events occurred along the CYST's outer edge, whereas for ISEA2, many of the additional CELL creation and DEATH events occurred within the LUMEN. The CELL creation events within LUMENS were enabled by the Axiom 4 action: create MATRIX between two CELLS. Blocking Axiom 4 use blocks almost all CELL creation events within LUMENS and promotes LUMEN clearance (not shown).

### Dysregulation of Axiom 6 (oriented CELL creation)

Oriented cell division is central to multicellular morphogenesis [25-27]. Matrix contact and cell adhesions play an important role in determining the orientation of the division axis in vitro [28,29]. Similar to its in vitro counterpart, CELL creation from Axiom 6 was oriented (not random). We dysregulated Axiom 6 by allowing the decision-making CELL to place a new CELL in a randomly selected MATRIX location, rather than selecting one that maximizes CELL contact.

**Figure 8:**
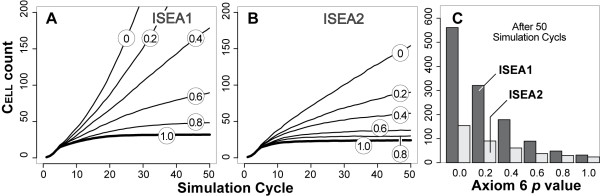
**Dysregulation of Axiom 6 and its effect on ISEA CULTURE growth**. Axiom 6 dictates oriented placement of a newly created CELL. It is placed at an adjacent MATRIX position that maximizes its number of CELL neighbors. CELLS followed Axiom 6 with a parameter-controlled probability, *p*. Otherwise, the CELL copy replaced a randomly selected MATRIX neighbor without regard for CELL neighbor number. Doing so changed ISEA growth and structural characteristics. (A-B) CELL count increased monotonically with the severity of dysregulation. Compared to ISEA1 growth (A), ISEA2 growth was affected less for every dysregulation level. (C) CELL count after 50 simulation cycles showed marked differences between ISEA1 and ISEA2 that increased with the severity of dysregulation.

We ran simulations with Axiom 6's *p *ranging from 0 to 1, and recorded changes in CULTURE growth and morphology along with CELL activity patterns. The overall results are shown in Figure 8. CULTURE growth rate and CELL count after 50 simulation cycles increased monotonically with Axiom 6 dysregulation. The changes were less dramatic than those observed following Axiom 5 dysregulation, and there were marked differences between dysregulated ISEA1 and ISEA2 CULTURE growth. ISEA2 was less susceptible to disoriented placement of a newly created CELL. Mean CELL count in ISEA2 CULTURES was always smaller than that for ISEA1 at every tested dysregulation level.

Dysregulating Axiom 6 using *p *= 0.8 and 0.6 increased CELL DEATH and CELL PROLIFERATION activities of ISEA2 less than ISEA1 (Figure 6). CELL DEATH events were offset by an approximately equal number of CELL creation events, and that was consistent with the observation that LUMEN-entrapped CELLS underwent cycles of CELL creation and DEATH.

Inspection of Figure 7C, D shows that the morphological irregularities resulting from a given degree of Axiom 6 dysregulation were less pronounced than from a corresponding degree of Axiom 5 dysregulation. For ISEA1, the morphology change produced by a degree of Axiom 6 dysregulation was very similar to that caused by a lesser degree of Axiom 5 dysregulation. ISEA1 structures produced using dysregulated Axiom 6 contained a larger fraction of POLARIZED CELLS than did corresponding Axiom 5 dysregulated structures, and so the former changed more slowly as simulations progressed. For ISEA2, because all CELL DEATH axioms were always followed, there was less LUMEN filling when Axiom 6 was dysregulated, compared to when Axiom 5 was disrupted to the same degree. As noted above, ISEA2 LUMEN filling was enabled by Axiom 4. Blocking it severely restrained and often eliminated formation of INTRALUMINAL CELL CLUSTERS.

### Dynamic phenotype

**Figure 9:**
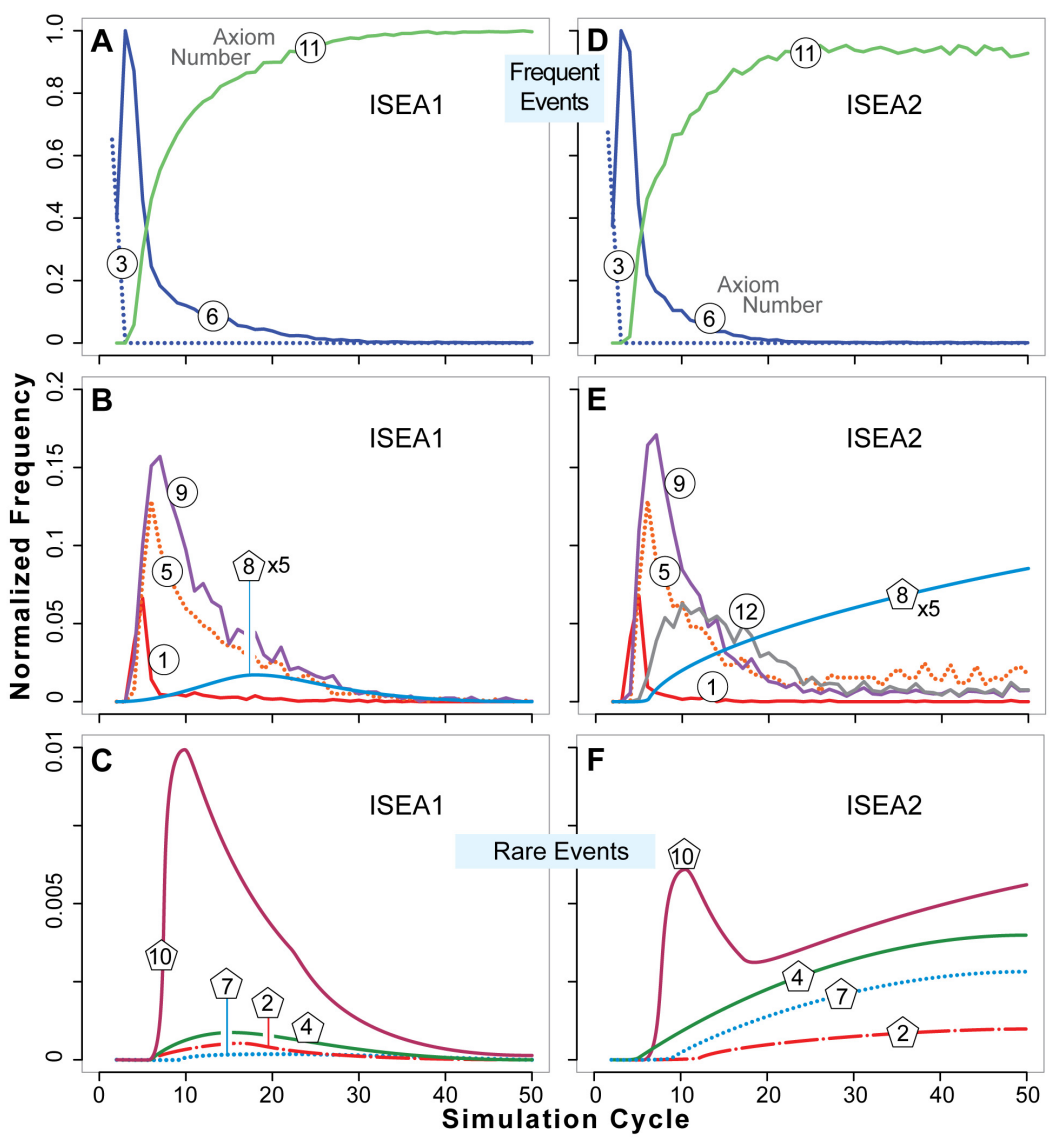
**Dynamic phenotype: axiom usage by ISEA1 and ISEA2**. Normalized axiom use frequencies are plotted versus simulation cycle. Left panels (A-C): ISEA1 use frequencies. Right panels (D-F): ISEA2 use frequencies. Top: axioms used most frequently. Middle: moderate use events. Bottom: Rare axiom use events. Axiom numbers in circles: the curves are normalized use frequencies averaged over 100 Monte Carlo runs. Axiom numbers in pentagons: the variance in average use frequency for the rarely used axioms was large; for clarity, trend lines are shown. In B and E, trend lines for Axiom 8 usage are magnified by a factor of 5. Raw data are provided in additional file 1: Supplemental Material. As simulations progressed and CYSTS matured, Axiom 11 (do nothing) was executed most frequently.

Figure 9 presents dynamic phenotype: the normalized frequency of axiom use by both ISEA1 and ISEA2. The CYSTOGENESIS mechanism at any stage in the process is the set of all events occurring within that interval. It is clear from Figure 9 that there is no specific CYSTOGENESIS mechanism. From start to the end of a simulation or until a stable structure forms, the mechanism evolves. How it evolves is a feature of that analogue's dynamic phenotype. Use patterns were similar for those axioms common to both analogues and that were used most frequently (1, 3, 5, 6, 9, and 11). Major differences were evident only for the less frequently used axioms (2, 4, 7, 8, and 10). As noted earlier, enabling CELL movement (Axiom 12) had an unanticipated consequence: it enabled the occasional formation of long-lived, small islands of CELLS within a LUMEN. Once a unit of MATRIX was formed, CELLS within a LUMEN could move and that gave rise to preconditions for creation of new CELLS as well as CELL DEATH. The process can continue for an extended interval and that accounts for the very low frequency of use of Axioms 2, 4, 7, and 8 by ISEA2. Note that when CELLS are trapped within an otherwise stable CYST, those INTRALUMINAL events are the only events. For that simulation, their relative use frequencies are large, and it is those values that are averaged with the values from other simulations, which are typically zero.

If nutrient levels within lumens are less than outside the cyst, then intraluminal cell division may not be sustainable. Furthermore, under 3D culture conditions, there is no direct evidence of matrix production by MDCK cells trapped within early-stage lumens during cystogenesis. It is noteworthy that by simulation cycle 50, when Axiom 4 is blocked, ISEA2's use frequency of axioms 2, 7, 8 and 10 drops to zero (not shown): ISEA2's axiom frequency of use pattern becomes similar to that of ISEA1.

**Figure 10:**
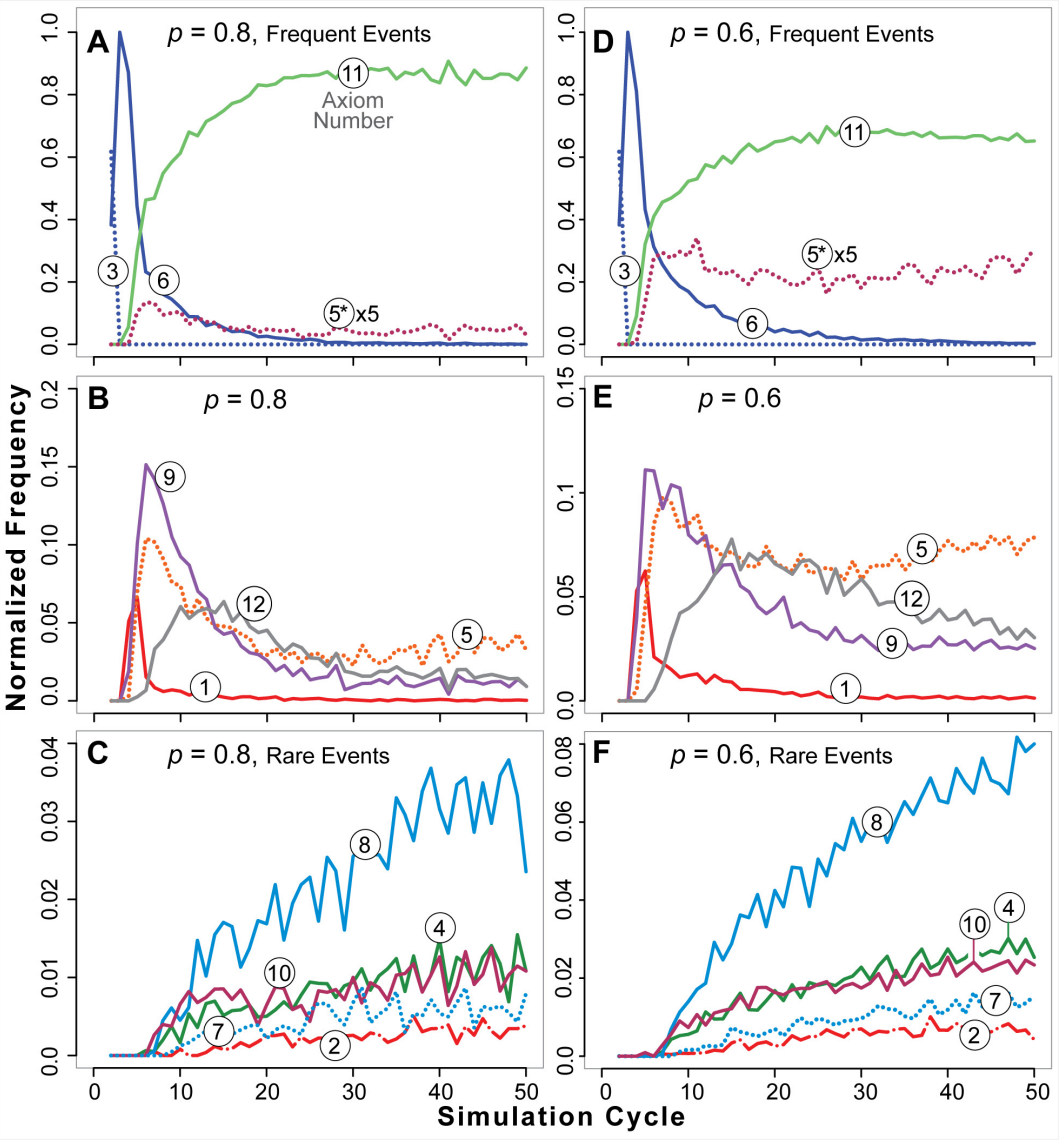
**Axiom usage by ISEA2 during partial Axiom 5 dysregulation**. Normalized axiom use frequencies are plotted versus simulation cycle as in Figure 9. Axiom numbers in circles are shown for each curve. *: dysregulated action. Left panels (A-C): *p *= 0.8. Right panels (D-F): *p *= 0.6. Top: axioms used most frequently. Middle: moderate use events. Bottom: Rare axiom use events. The curves are normalized use frequencies averaged over 100 Monte Carlo runs. In A and B, Axiom 5* usage frequencies are magnified by a factor of 5.

**Figure 11:**
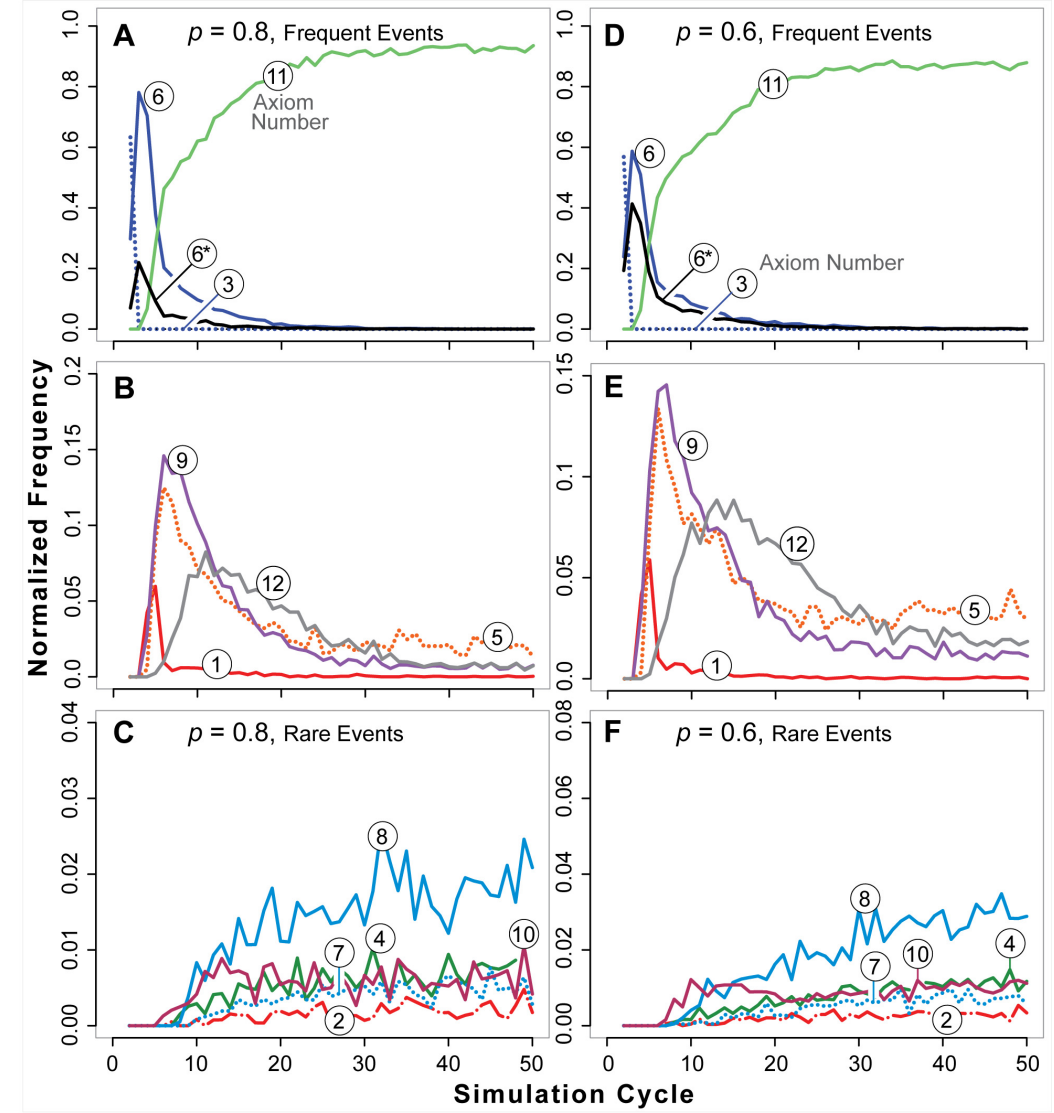
**Axiom usage by ISEA2 during partial Axiom 6 dysregulation**. Normalized axiom use frequencies are plotted versus simulation cycle as in Figures 9 and 10. Axiom numbers in circles are shown for each curve. *: dysregulated action. Left panels (A-C): *p *= 0.8. Right panels (D-F): *p *= 0.6. Top: axioms used most frequently. Middle: moderate use events. Bottom: Rare axiom use events. The curves are normalized use frequencies averaged over 100 Monte Carlo runs.

Axiom dysregulation changed dynamic phenotype. Additional records for dysregulating Axioms 5 and 6 are provided in additional file 1: Supplemental Material for both ISEA1 and ISEA2. Because trends are similar for ISEA1 and ISEA2, we present in Figures 10 and 11 selected results for ISEA2. Figure 10 shows ISEA2 axiom use frequencies for Axiom 5 *p *= 0.8 and 0.6. The major consequence was reduction in Axiom 11 usage (do nothing: mandates achieved). That decline was mirrored by the rise in Axiom 5* (dysregulated action) usage, which remained relatively constant after five simulation cycles. In parallel, the use patterns for all other axioms changed relative to their *p *= 1 patterns. Even though only Axiom 5 was disrupted occasionally, all ISEA2 operating principles were impacted to some extent: the entire dynamic phenotype changed. However, the morphological consequences for *p *= 0.8 were difficult to detect: except for a tendency to be larger, most stabilized CYSTS were indistinguishable from those formed when *p *= 1. The potential morphological consequences of relaxing Axiom 5's *p *by 20% were thwarted by small shifts in the use frequencies of all other axioms. This observation suggests that the networked nature of ISEA2 axiom usage acts to buffer the consequences of small disruptions of any one operating principle.

Both the morphological and dynamic phenotypic consequences of Axiom 6 dysregulation were less dramatic than those of Axiom 5. They were also less dramatic in ISEA2 than in ISEA1. Reducing *p *led to larger structures that eventually stabilized (Figure 8B) and to more CELLS being trapped within occasional LUMENS (Figure 7D). Comparison of Figures 10 and 11 reveals that the influence of Axiom 6 disruption was also less significant than that of disrupting Axiom 5 to the same degree. For *p *= 0.8 and 0.6, the activities of CELLS trapped in LUMENS were primarily responsible for increased axiom use after about 20 simulation cycles. When Axiom 4 was blocked (not shown), those axiom use frequencies diminished considerably making an increased CYST size the primary consequence of Axiom 6 disruption.

## Discussion

We detailed a computational approach to build and test plausible hypotheses of in vitro dynamic phenotype. The newly developed framework enabled MDCK cell-mimetic analogues to function as autonomously as feasible for software agents. Axiomatic operating principles enabled ISEA2 CELLS to consistently produce convex CYSTS under simulated 3D embedded culture condition. Measures of axiom use during CYSTOGENESIS provided a detailed description of ISEA2 dynamic phenotype. Dysregulating key CELL DEATH and DIVISION axioms led to disorganized cystic forms that were reminiscent of the in vitro tumor reconstruction phenotype. Unexpectedly, ISEA2's drive for convexity made it less susceptible to, or more robust against, the dysregulation of either axiom when compared to its predecessor, ISEA1. It will be interesting to learn if the mechanisms underlying epithelial cyst convexity in cultures contribute to robustness against comparable interventions. In addition, occasional disruption of one activity in a minority of CELLS, as in Figures 10 and 11, had consequences for the system (e.g., altered CYST morphology) and for all other normal behaving CELLS. The average axiom use patterns of all other CELLS changed. Upon reflection, the observation could be expected. The actions of all CELLS in a CLUSTER transforming into a CYST are networked in space and time. An action of one CELL can affect the action options of a nearby CELL at a future time. If a CELL occasionally malfunctions, it has measurable consequences, as shown in Figures 10 and 11. To the extent that the mappings in Figure 1 are accepted as valid, we can extend such observations to MDCK epithelial cells undergoing morphogenesis.

The results reaffirm that Axioms 5 and 6 play critical, dominant roles in determining the CYSTOGENESIS phenotype. Also, as noted in Results, Axiom 1 was essential for initial LUMINAL SPACE creation, and completely blocking its use had a detrimental effect on CULTURE morphology. On the other hand, Axioms 2–4 and 7 were nonessential for CYSTOGENESIS in EMBEDDED CULTURE. Dysregulating or simply deleting the axioms did not patently alter the CYSTOGENESIS phenotype. However, that does not mean that the axioms were not parsimonious: they were essential to achieving targeted attributes of the other CULTURE types—SUSPENSION, SURFACE, and OVERLAY—from [9]. Whether a similar relationship holds true for their biological counterpart is unknown. However, it is clear that MDCK cells under different culture conditions use somewhat different cell mechanisms depending on the specific culture condition, which leads to different culture phenotypes [2,30-32].

While reasonable mappings can be established from ISEA to MDCK and MCF-10A mammary epithelial cell phenotypes [16], ISEA axioms may not map well to other epithelial cell types and culture systems. For example, in AT II cell cultures, cyst structures develop by a mechanism that involves neither cell death nor proliferation [33]. Alveolar-like cysts form by cell migration and aggregation, in contrast to how cysts typically develop in MDCK cell cultures. Those differences are mirrored in validated CELL axiom specifications of the ISEAs and AT II analogues. Unlike the ISEA CELLS, the AT II analogue [6] lacks CELL DEATH and PROLIFERATION action options. They form CYSTS exclusively by spatial rearrangement. Notwithstanding those differences, their stable form similarities suggest common mandates. For instance, ISEA and AT II analogues do exhibit a common, essential feature: CELLS strive to achieve and maintain lateral CELL-CELL contacts. Additional insight is anticipated when 2D simulations are expanded to 3D.

Cell processes work together in ways that give rise to effective mandates that normal epithelial cells appear to follow. Each mandate is assumed a consequence of the interoperation of genetics and environmental factors. How specific cell actions contribute to these mandates is unclear. However, tracing CELL activities during ISEA2 simulations makes clear how their mandates, the targeted attributes, are achieved. That clarity provides insight into and plausible explanations of MDCK's morphogenic phenomena. Because ISEA components and mechanisms are coarse-grained, one ISEA2 axiom may map to many fine-grain MDCK processes. Iterative refinement of ISEA2 so that it achieves an expanded set of MDCK attributes will improve and concretize the mappings from analogue to MDCK cultures, potentially creating new knowledge. Mappings from specifics of MDCK cultures (complex) to analogue (simplified), however, will always be ambiguous, a property of all referent-model pairs.

Moving forward, we suggest the following iterative refinement protocol. It was used successfully herein and in previous studies [4-6,9,34]. The protocol supports adhering to the guideline of parsimony which is important when building a complex model. It is straightforward and so can be used for refinement of any mechanistically focused, agent-based biomimetic analogue. Basic steps are: 1) start with a small but diverse set of in vitro attributes, static and dynamic. They are the initial targeted attribute list. 2) Posit coarse-grained, discrete mechanisms, requiring as few components as is reasonable, that may generate analogous phenomena. 3) Instantiate (represent an abstraction by a concrete software instance) analogue components and mechanisms. 4) Conduct experiments to measure a variety of phenomena generated during execution. So doing establishes the degree of in silico-in vitro phenotype overlap, and lack thereof. 5) Achieve a degree of validation by satisfying a prespecified level of similarity between in silico and targeted in vitro attributes. 6) Add one or more new attributes (measurable phenomena) to the targeted list until the analogue in step 5 is falsified. Added attributes need to be at a similar level to and sufficiently close to those already present so that it seems feasible to achieve the expanded attribute list with as little component reengineering as possible. Once the analogue in step 5 is falsified, return to step 2.

The nature and organization of software components within the ISEA framework, as illustrated in Figure 2, were designed to facilitate iterative refinement of everything on the right side of Figure 1. That process can concretize each of the mappings from ISEA to MDCK counterparts. As the process continues, following each round of validation, more of what we know or think we know becomes instantiated in the analogue. After many such rounds, the analogue will mature as instantiated, working hypotheses of how MDCK cystogenesis and pathologic transformations occur. At that stage, it will have become an extensible, interactive instantiation of available biological knowledge about mechanisms and processes. It will have become an executable knowledge embodiment. To achieve that vision, it is essential that biomimetic components function (quasi-) autonomously, all or part of the time. That is why CELLS are agents. Everything that a CELL needs to function (in a specified software environment) is contained within its code. Absent that property, the mappings from ISEA to MDCK cystogenesis mechanisms are not concretizable, and so the mappings from ISEA to MDCK operating principles are forced to remain conceptual.

Finally, axiom use results show that at the same time, different CELLS within the same CULTURE are engaged in quite different activities. The same is true in vitro; one MDCK cell can be moving actively relative to its attached neighbors while another is undergoing anoikis, and yet another is initiating division. Simultaneously, polarized cells that have achieved their mandates may begin downregulating processes used earlier. It follows that the ensemble of molecular biology details, such as gene and protein expression levels, which enable those different activities will themselves be different. Patterns detected in gene and protein expression data averaged over all cells in an active cyst may have little scientific value in answering such questions as these. When and how does an epithelial cell choose to switch from one activity to another? Why does it choose one action rather than another? Are several action options always available to each cell? Obtaining plausible answers to these and related questions is essential to achieving deeper insight into epithelial morphogenesis and early cancer progression. As demonstrated, the class of models presented herein provides a rigorous platform to hypothesize, challenge, and refine plausible answers. The causal chain of events responsible for most simulated behaviors can be explored in detail, and assessments made as to whether critical events are biotic (supportable by in vitro evidence) or not.

## Conclusion

The approach described herein provided for a hypothesis—a theory—of how the collective consequences of individual MDCK cell actions might give rise to systemic in vitro phenotype. The causal chain of events responsible for most ISEA behaviors could be explored in detail, and assessments could be made of their relative roles during simulation. Having that capability enabled us to develop a detailed dynamic ISEA phenotype. The MDCK embedded culture counterpart is problematic to obtain using state-of-the-art in vitro methods. We expect future rounds of model refinement and validation will strengthen in silico-to-in vitro mappings, thus providing a viable strategy to gain deeper insight into the mechanistic basis of epithelial cystogenesis, morphogenesis, and in vitro transformations.

## Competing interests

The authors declare that they have no competing interests.

## Authors' contributions

SK and CH conceived the idea. SK designed and performed the experiments. SP participated in the design and implementation. SK, KM, JD, and CH analyzed the experiment results. SK and CH wrote the paper with input from coauthors. All authors read and approved the final manuscript.

## Supplementary Material

Additional File 1 **Supplemental Material**. Provided are complete, raw axiom usage data. Click here for file
