# Supplementary material for: Computational investigation of epithelial cell dynamic phenotype in vitro
Source: Theor Biol Med Model. 2009 May 28;6:8. doi: 10.1186/1742-4682-6-8 (PMC2696420; doi:10.1186/1742-4682-6-8)
Supplement: Additional File 1 — Supplemental Material. Provided are complete, raw axiom usage data. [file 1742-4682-6-8-S1.pdf]

Supplementary Material

for

Computational Investigation of Epithelial Cell Dynamic Phenotype In Vitro

Sean HJ Kim, Sunwoo Park, Keith Mostov, Jayanta Debnath, C Anthony Hunt

Table S1. Supplementary figures

Figure	Analogue	Dysregulated Axiom	Description
S1	ISEA 1, 2	5	Axiom use frequencies (frequent, moderate, rare use events) for *p* = 1, 0.8, and 0.6
S2	ISEA 1, 2	5	
S3	ISEA 1, 2	5	
S4	ISEA 1, 2	6	Axiom use frequencies (see above)
S5	ISEA 1, 2	6	
S6	ISEA 1, 2	6	
S7	ISEA 1, 2	5 and 6	Axiom use frequencies when both Axioms' *p* = 1, 0.8, and 0.6
S8	ISEA 1, 2	5 and 6	
S9	ISEA 1, 2	5 and 6	

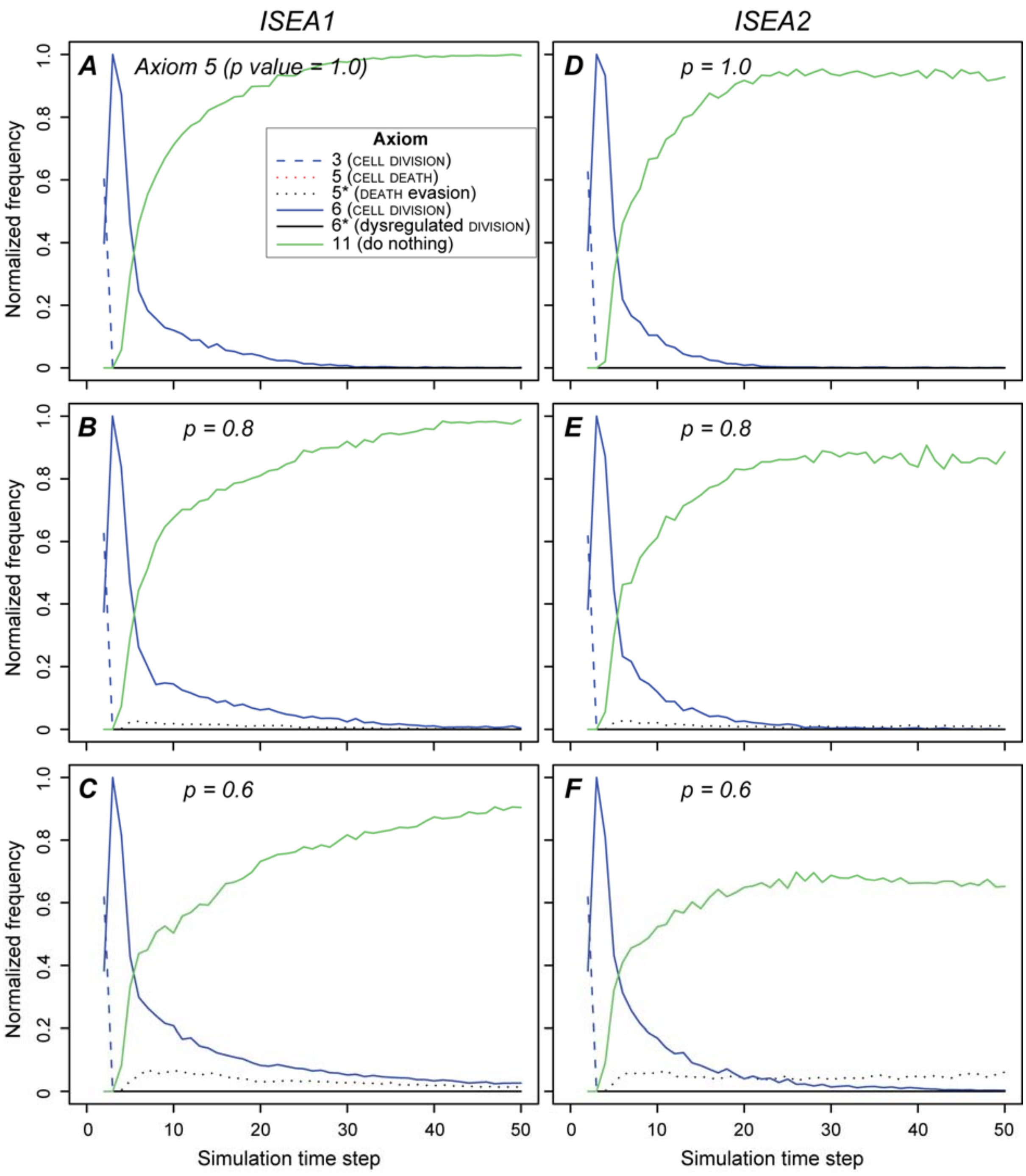

Figure S1. Frequently used axiom usage by ISEA1 and ISEA2 during Axiom 5 dysregulation

Normalized axiom use frequencies are plotted versus simulation cycle. One simulation cycle maps to ~ 12 hours of MDCK culture. Axioms 3, 5, 5* (dysregulated form), 6, 6*, and 11 are shown. (A-C) Shown are ISEA1 axiom use frequencies. (D-F) ISEA2 axiom use frequencies are shown. The curves are normalized use frequencies averaged over 100 Monte Carlo runs.

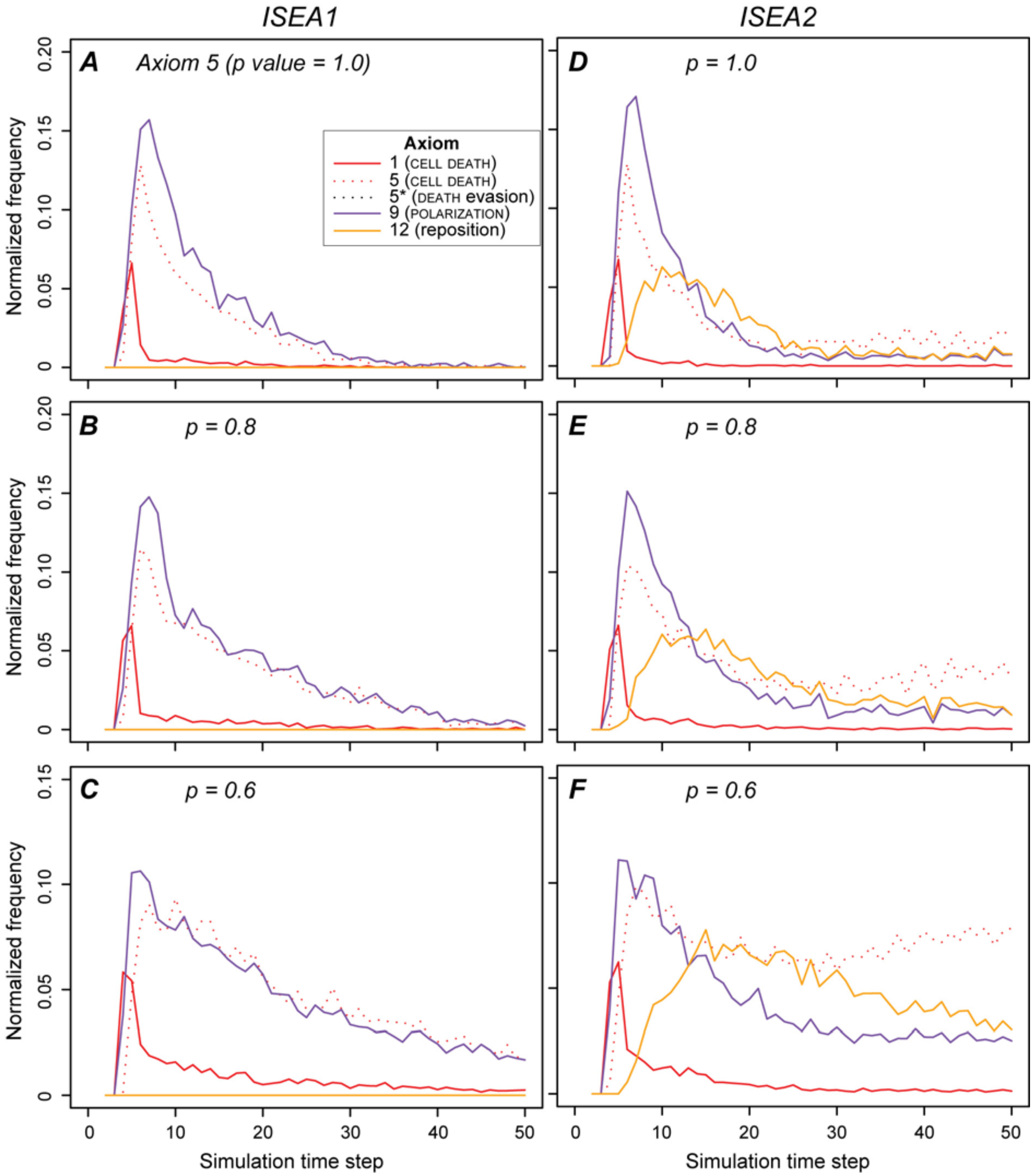

Figure S2. Moderately used axiom usage by ISEA1 and ISEA2 during Axiom 5 dysregulation

Normalized axiom use frequencies are plotted versus simulation cycle. Axioms 1, 5, 9, and 12 are shown. One simulation cycle maps to ~ 12 hours of MDCK culture. (A-C) Shown are ISEA1 axiom use frequencies. (D-F) ISEA2 axiom use frequencies are shown. The curves are normalized use frequencies averaged over 100 Monte Carlo runs.

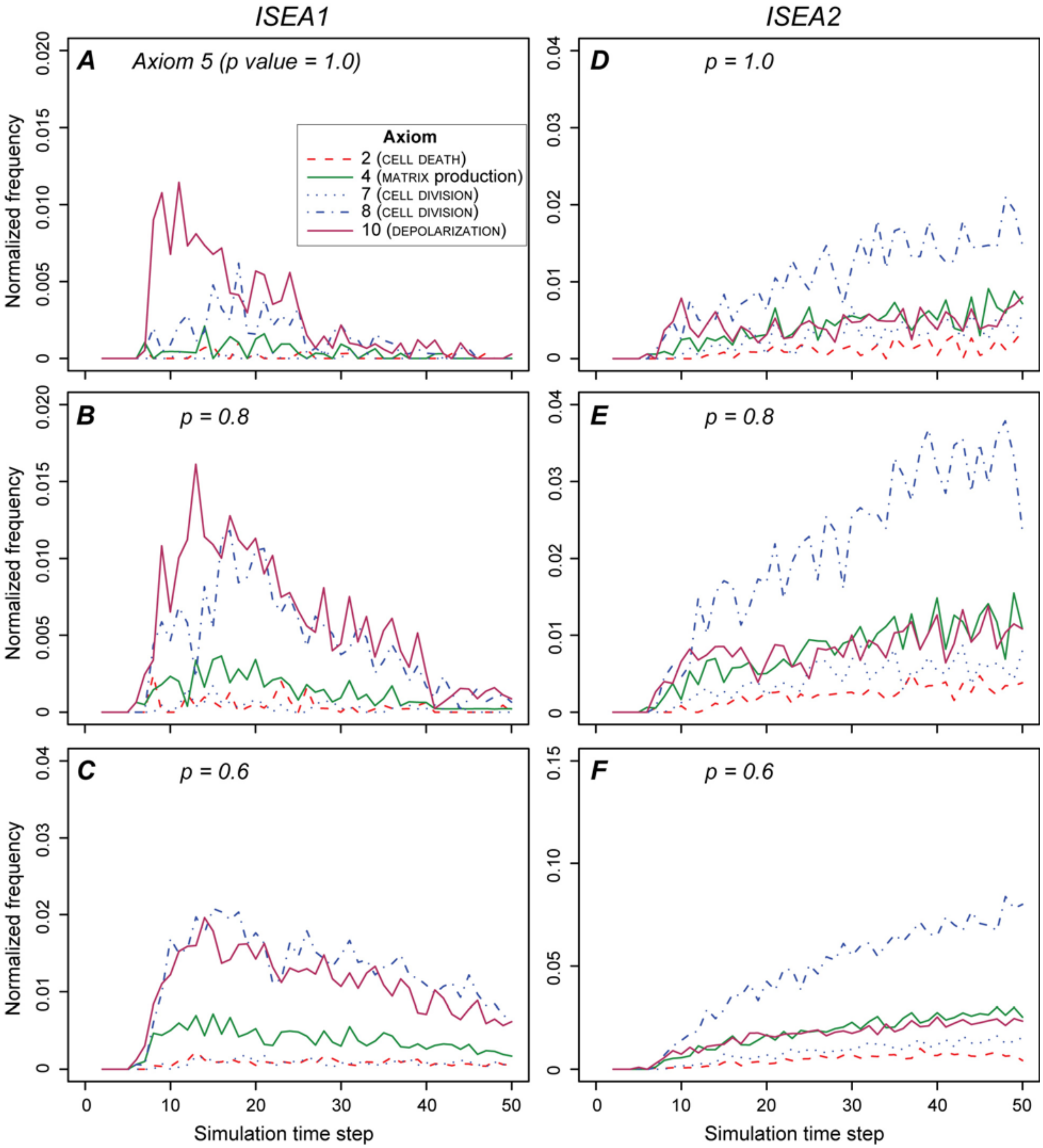

Figure S3. Rare use axiom usage by ISEA1 and ISEA2 during Axiom 5 dysregulation

Normalized axiom use frequencies are plotted versus simulation cycle. Axioms 2, 4, 7, 8, and 10 are shown. One simulation cycle maps to ~ 12 hours of MDCK culture. (A-C) Shown are ISEA1 axiom use frequencies. (D-F) ISEA2 axiom use frequencies are shown. The curves are normalized use frequencies averaged over 100 Monte Carlo runs.

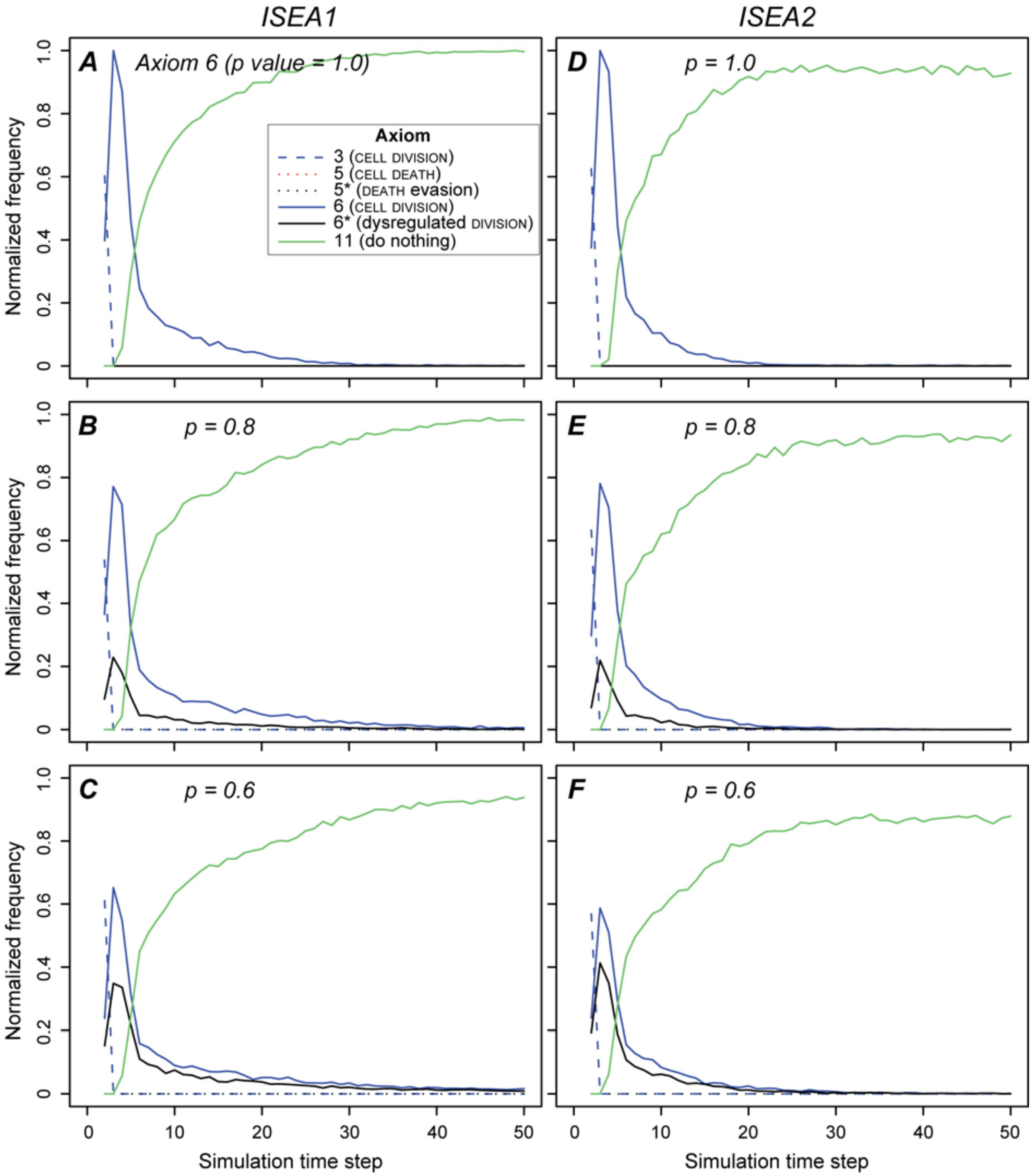

Figure S4. Frequently used axiom usage by ISEA1 and ISEA2 during Axiom 6 dysregulation

Normalized axiom use frequencies are plotted versus simulation cycle. One simulation cycle maps to ~ 12 hours of MDCK culture. Axioms 3, 5, 5* (dysregulated form), 6, 6*, and 11 are shown. (A-C) Shown are ISEA1 axiom use frequencies. (D-F) ISEA2 axiom use frequencies are shown. The curves are normalized use frequencies averaged over 100 Monte Carlo runs.

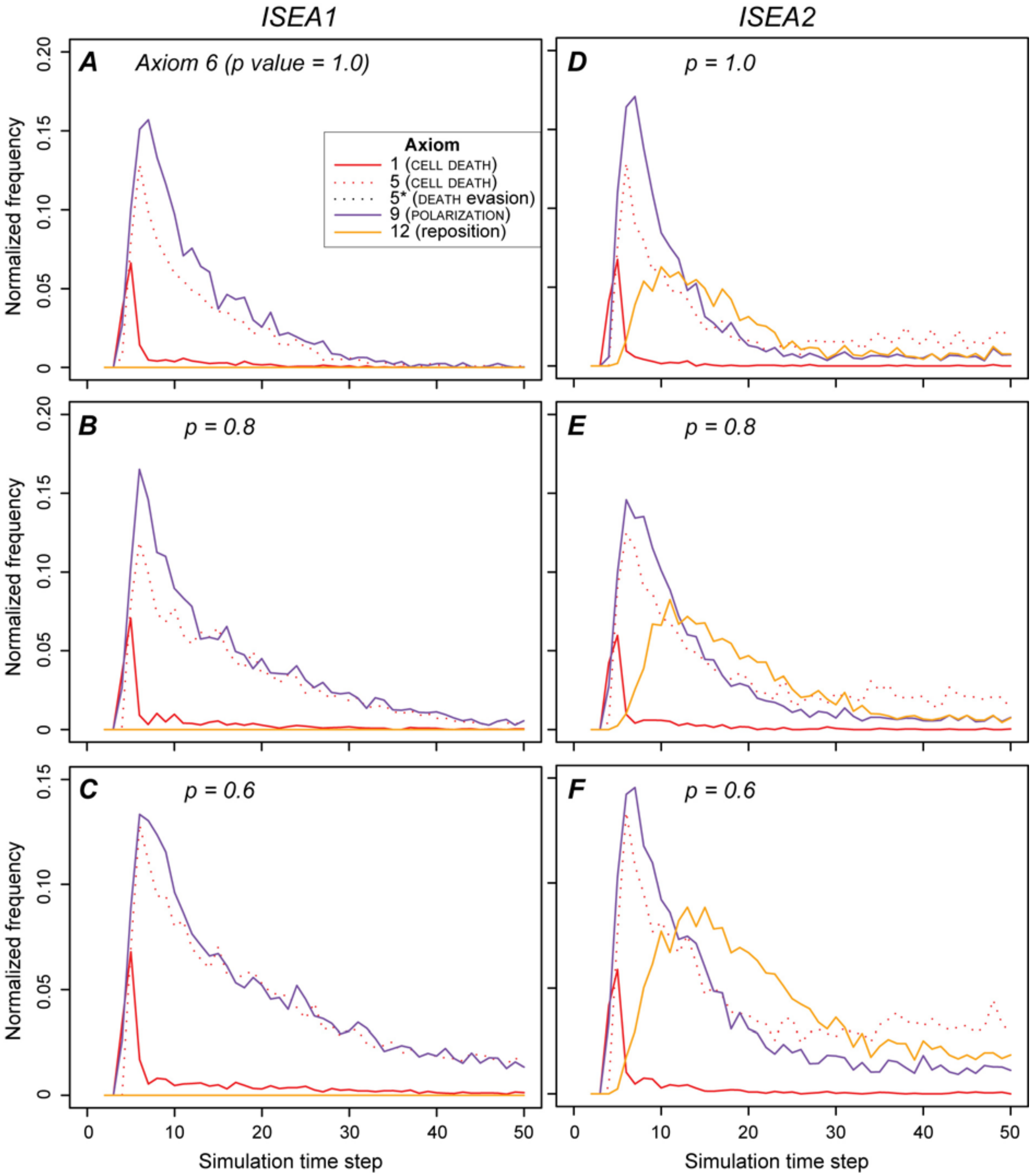

Figure S5. Moderately used axiom usage by ISEA1 and ISEA2 during Axiom 6 dysregulation

Normalized axiom use frequencies are plotted versus simulation cycle. Axioms 1, 5, 9, and 12 are shown. One simulation cycle maps to ~ 12 hours of MDCK culture. (A-C) Shown are ISEA1 axiom use frequencies. (D-F) ISEA2 axiom use frequencies are shown. The curves are normalized use frequencies averaged over 100 Monte Carlo runs.

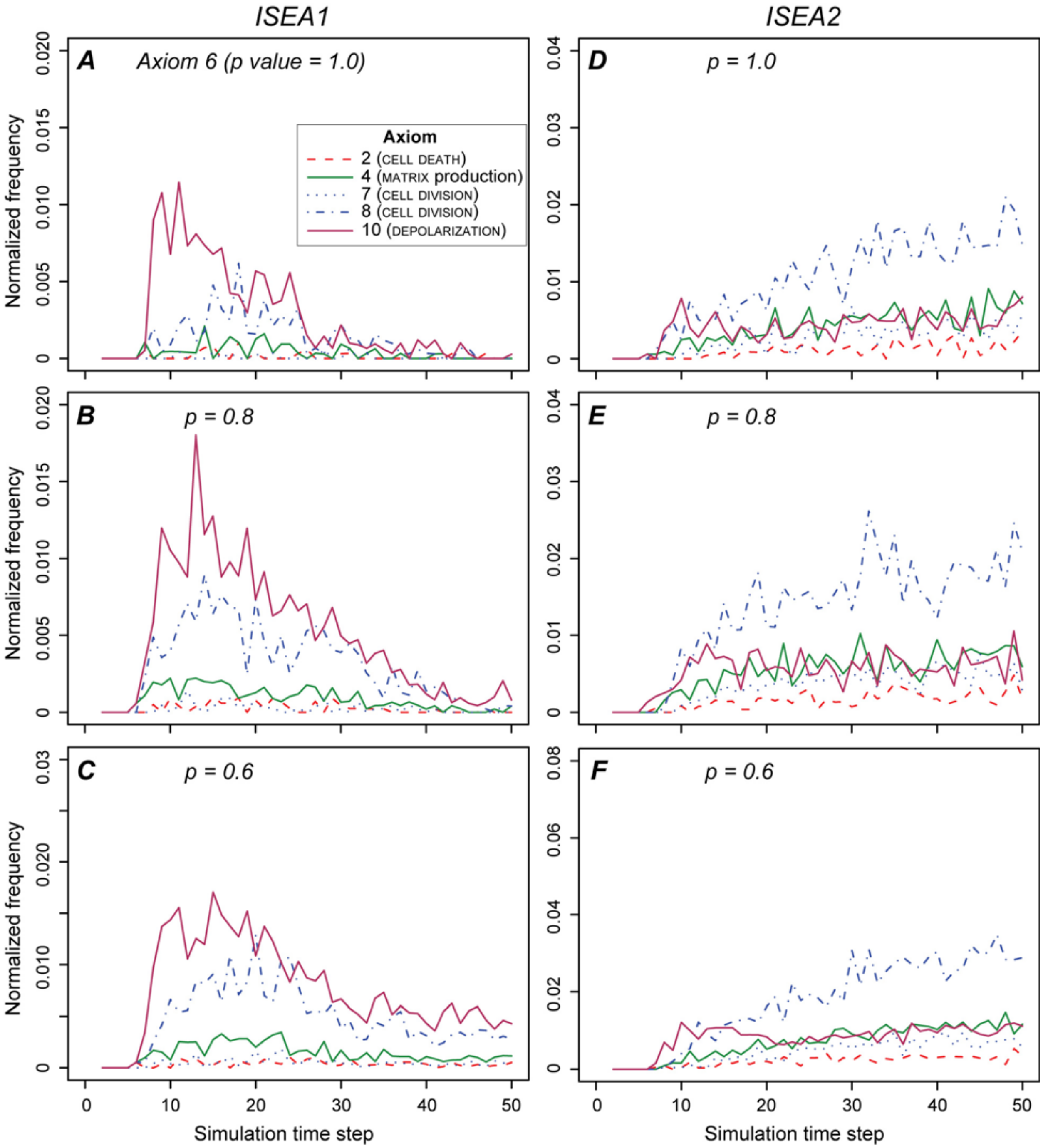

Figure S6. Rare use axiom usage by ISEA1 and ISEA2 during Axiom 6 dysregulation
Normalized axiom use frequencies are plotted versus simulation cycle. Axioms 2, 4, 7, 8, and 10 are shown. One simulation cycle maps to ~ 12 hours of MDCK culture. (A-C) Shown are ISEA1 axiom use frequencies. (D-F) ISEA2 axiom use frequencies are shown. The curves are normalized use frequencies averaged over 100 Monte Carlo runs.

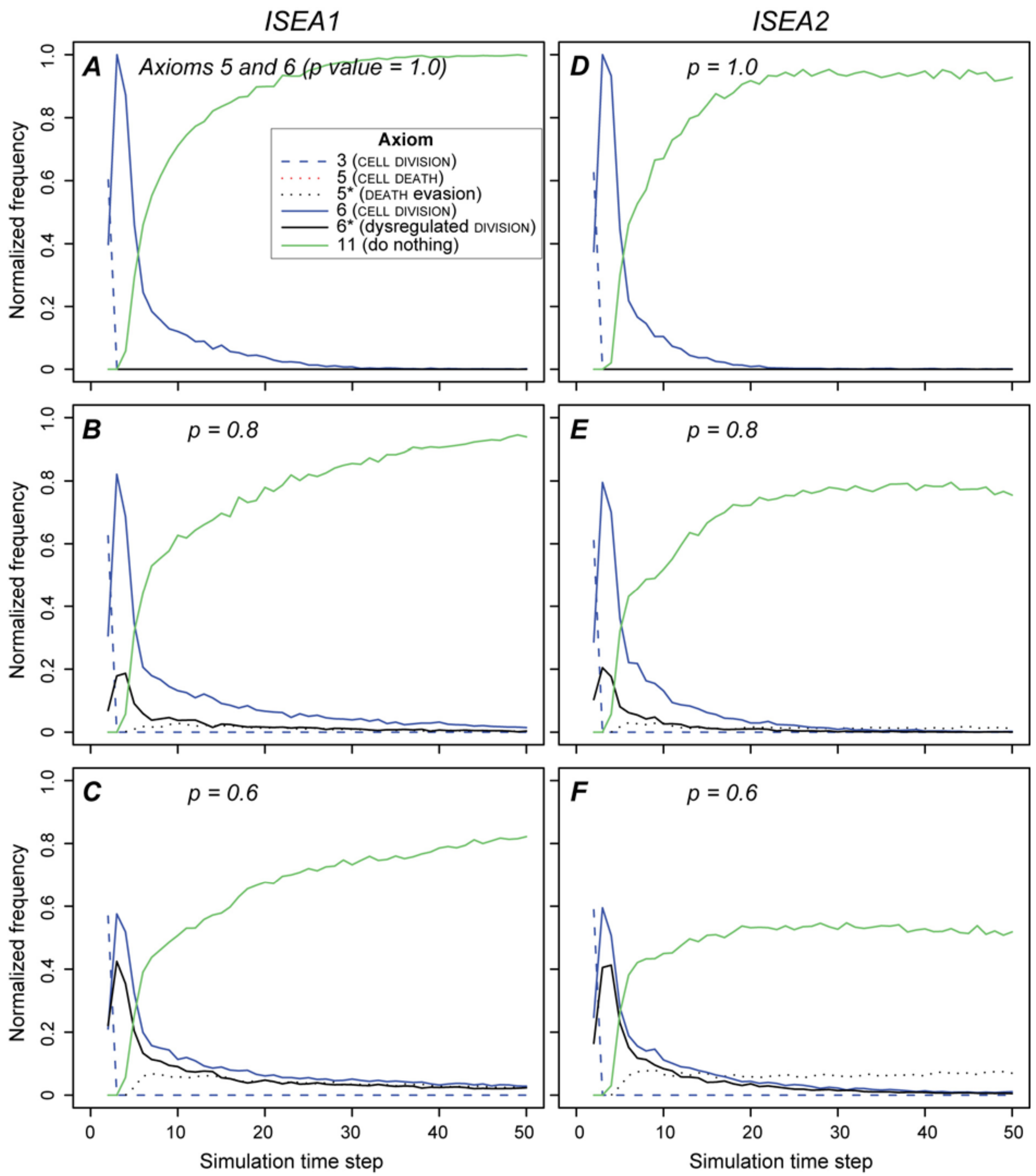

Figure S7. Frequently used axiom usage by ISEA1 and ISEA2 during simultaneous dysregulation of Axioms 5 and 6

Normalized axiom use frequencies are plotted versus simulation cycle. One simulation cycle maps to ~ 12 hours of MDCK culture. Shown are Axioms 3, 5, 5* (dysregulated form), 6, 6*, and 11. (A-C) Shown are ISEA1 axiom use frequencies. (D-F) ISEA2 axiom use frequencies are shown. The curves are normalized use frequencies averaged over 100 Monte Carlo runs.

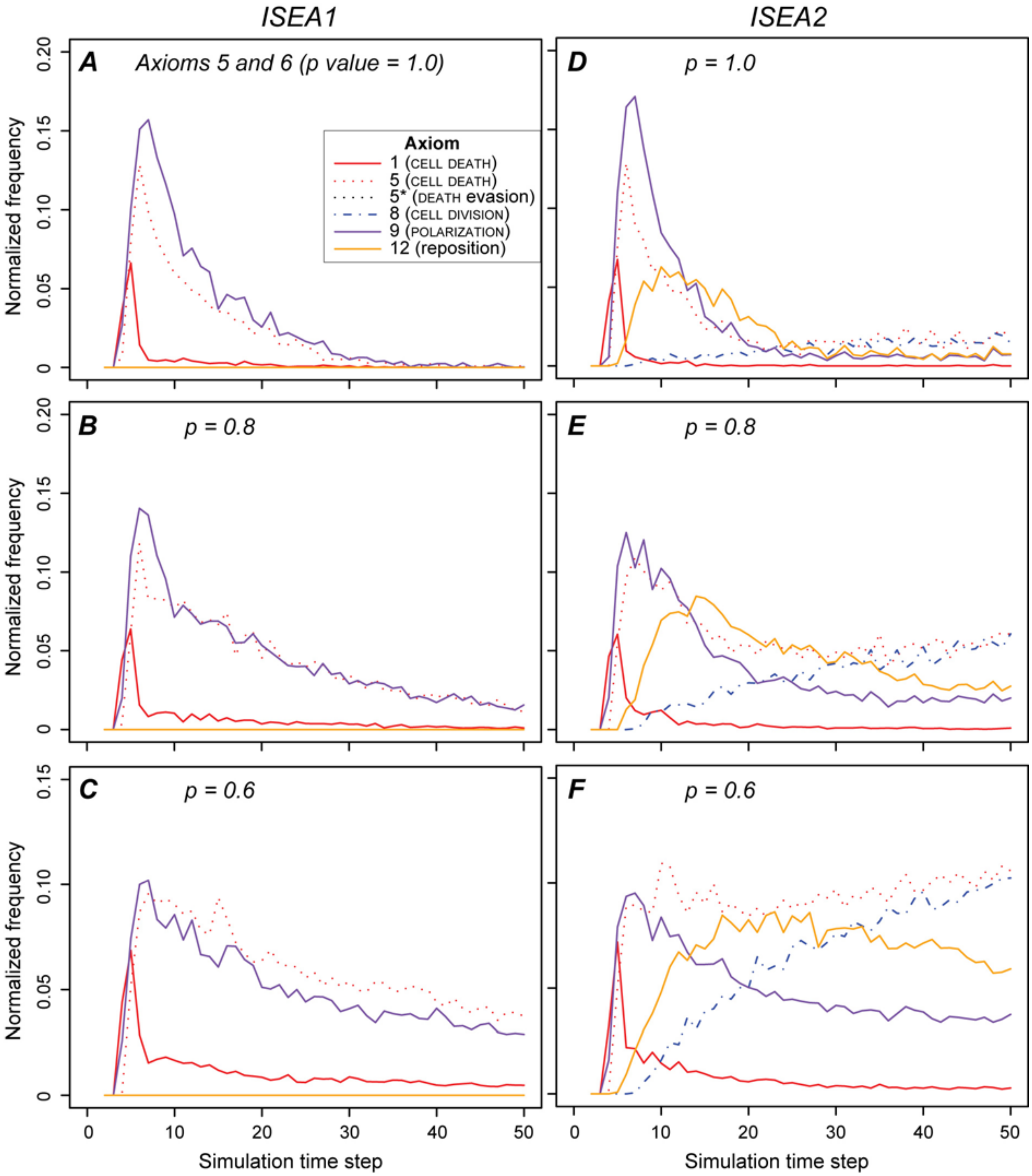

Figure S8. Moderately used axiom usage by ISEA1 and ISEA2 during simultaneous dysregulation of Axioms 5 and 6

Normalized axiom use frequencies are plotted versus simulation cycle. Axioms 1, 5, 5* (dysregulated form), 8, 9, and 12 are shown. One simulation cycle maps to ~ 12 hours of MDCK culture. (A-C) Shown are ISEA1 axiom use frequencies. (D-F) ISEA2 axiom use frequencies are shown. The curves are normalized use frequencies averaged over 100 Monte Carlo runs.

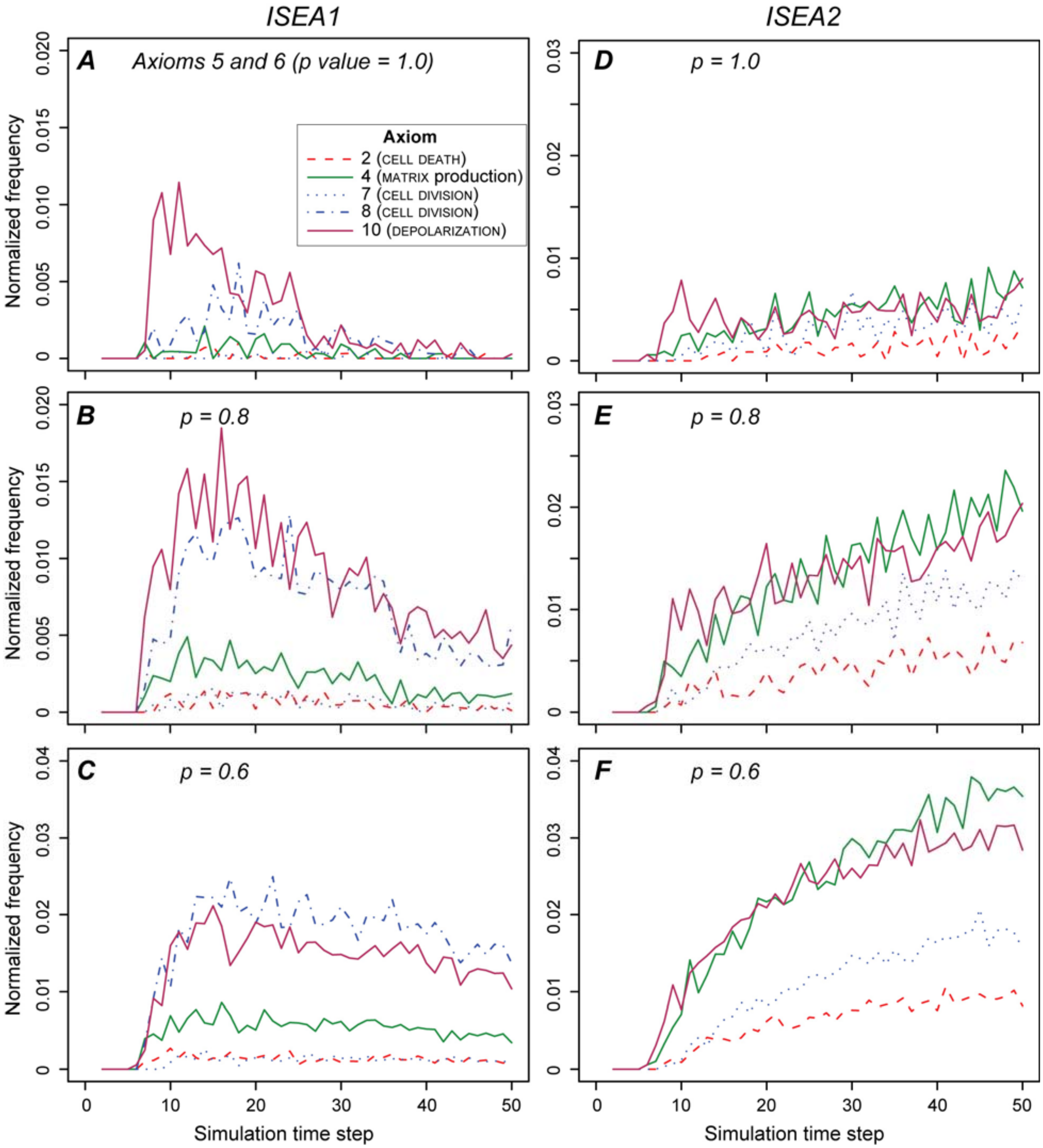

Figure S9. Rare use axiom usage by ISEA1 and ISEA2 during simultaneous dysregulation of Axioms 5 and 6

Normalized axiom use frequencies are plotted versus simulation cycle. Axioms 2, 4, 7, 8, and 10 are shown. One simulation cycle maps to ~ 12 hours of MDCK culture. (A-C) Shown are ISEA1 axiom use frequencies. (D-F) ISEA2 axiom use frequencies are shown. The curves are normalized use frequencies averaged over 100 Monte Carlo runs.
